# Characterizing Adult Cochlear Supporting Cell Transcriptional Diversity Using Single-Cell RNA-Seq: Validation in the Adult Mouse and Translational Implications for the Adult Human Cochlea

**DOI:** 10.3389/fnmol.2020.00013

**Published:** 2020-02-05

**Authors:** Michael Hoa, Rafal Olszewski, Xiaoyi Li, Ian Taukulis, Shoujun Gu, Alvin DeTorres, Ivan A. Lopez, Fred H. Linthicum Jr., Akira Ishiyama, Daniel Martin, Robert J. Morell, Matthew W. Kelley

**Affiliations:** ^1^Auditory Restoration and Development Program, National Institute on Deafness and Other Communication Disorders, NIH, Bethesda, MD, United States; ^2^National Temporal Bone Laboratory at UCLA, UCLA School of Medicine, Los Angeles, CA, United States; ^3^Cellular and Molecular Biology of the Inner Ear Laboratory, UCLA School of Medicine, Los Angeles, CA, United States; ^4^Biomedical Research Informatics Office, National Institute of Dental and Craniofacial Research, NIH, Bethesda, MD, United States; ^5^Genomics and Computational Biology Core, National Institute on Deafness and Other Communication Disorders, NIH, Bethesda, MD, United States; ^6^Laboratory of Cochlear Development, National Institute on Deafness and Other Communication Disorders, NIH, Bethesda, MD, United States

**Keywords:** inner ear, supporting cell subtypes, smFISH, adult (MeSH), cell cycle, FACS, cochlea

## Abstract

Hearing loss is a problem that impacts a significant proportion of the adult population. Cochlear hair cell (HC) loss due to loud noise, chemotherapy and aging is the major underlying cause. A significant proportion of these individuals are dissatisfied with available treatment options which include hearing aids and cochlear implants. An alternative approach to restore hearing would be to regenerate HCs. Such therapy would require a recapitulation of the complex architecture of the organ of Corti, necessitating regeneration of both mature HCs and supporting cells (SCs). Transcriptional profiles of the mature cell types in the cochlea are necessary to can provide a metric for eventual regeneration therapies. To assist in this effort, we sought to provide the first single-cell characterization of the adult cochlear SC transcriptome. We performed single-cell RNA-Seq on FACS-purified adult cochlear SCs from the *Lfng^EGFP^* adult mouse, in which SCs express GFP. We demonstrate that adult cochlear SCs are transcriptionally distinct from their perinatal counterparts. We establish cell-type-specific adult cochlear SC transcriptome profiles, and we validate these expression profiles through a combination of both fluorescent immunohistochemistry and *in situ* hybridization co-localization and quantitative polymerase chain reaction (qPCR) of adult cochlear SCs. Furthermore, we demonstrate the relevance of these profiles to the adult human cochlea through immunofluorescent human temporal bone histopathology. Finally, we demonstrate cell cycle regulator expression in adult SCs and perform pathway analyses to identify potential mechanisms for facilitating mitotic regeneration (cell proliferation, differentiation, and eventually regeneration) in the adult mammalian cochlea. Our findings demonstrate the importance of characterizing mature as opposed to perinatal SCs.

## Introduction

Hearing loss impacts approximately 432 million adults worldwide (WHO Media Centre, 2018). For most of these individuals, the underlying cause of the auditory dysfunction is a loss of mechanosensory hair cells (HCs) in the cochlea. Existing data for humans, and all other mammals, strongly suggests that loss of cochlear HCs in adults is permanent (Doetzlhofer et al., 2006; White et al., 2006). In contrast, some non-mammalian vertebrates, such as avian species, are able to robustly and repeatedly regenerate HCs in response to injury. In these species, supporting cells (SCs), which surround the HCs, act as precursors which give rise to new HCs through both proliferative and non-proliferative mechanisms (Corwin and Cotanche, 1988; Ryals and Rubel, 1988; White et al., 2006; Warchol, 2011; Liu et al., 2012; Atkinson et al., 2015).

Studies utilizing both viral gene delivery and genetically-inducible mouse models have demonstrated some success in converting SCs into HCs in perinatal, but not adult, mouse models (Praetorius et al., 2010; Kelly et al., 2012; Liu et al., 2012, 2014; Staecker et al., 2014; Kuo et al., 2015). An exception to this is the recent work from Walters et al. (2017) demonstrating the formation of new HCs from adult cochlear SCs using a combination of forced expression of *Atoh1*, a HC master regulator gene, and deletion of *Cdkn1b*, a gene that controls cell cycle exit in SCs and HC precursors. But even in this case the HCs that were generated remained immature and, in some cases, showed signs of apoptotic cell death (Walters et al., 2017). These results suggest that our understanding of the genetic pathways that must be modulated to achieve a biological restoration of hearing remains limited.

While considerable effort has been devoted to understanding the genetic pathways that modulate HC formation, it is equally important to determine how cells are specified as SCs. As discussed, regenerated HCs will, most likely, be derived from SCs, which will require de- and then re-differentiation potentially similar to the process observed in avian species (Stone and Cotanche, 2007). The ability to accomplish SC-mediated HC regeneration in the adult organ of Corti is limited by barriers that remain to be characterized. A first step towards overcoming these barriers would be to generate expression profiles for specific SC types within the adult cochlea. Therefore, we sought to characterize the transcriptomes of adult cochlear SCs using single-cell RNA-sequencing. The results demonstrate that adult cochlear SCs are transcriptionally distinct from their perinatal counterparts. We identify adult cochlear SC-specific expression profiles and extensively validate these expression profiles through a combination of fluorescent immunohistochemistry and *in situ* hybridization co-localization in adult cochlear cross-sections and quantitative polymerase chain reaction (qPCR) from isolated adult cochlear SCs. To examine the relevance of these pathways for potential clinical applications, we demonstrate the expression of several novel, cell-type-specific markers using immunofluorescence on human temporal bones. Finally, we perform cell cycle pathway analyses on FACS-purified single adult SC transcriptomes to explore potential mechanisms to overcome adult SC quiescence.

## Materials and Methods

Key resources are provided in Table 1.

**Table 1:** Key resources.

Reagent or resource*	Source	Identifier
**Antibodies**
S100A6	R&D	AF4584
LCP1	Cell Signaling	3588
NOTCH1	Cell Signaling	3608
Acetylated tubulin (Tuba4a)	Sigma-Aldrich	T6793
S100A1	NeoMarkers	RB-044-A0
DSTN	Sigma	D8815
Calbindin	Santa Cruz	Sc-365360
**RNAScope *in situ* probes**		
Mm-S100a6	Advanced Cell Diagnostics	412981
Mm-Lcp1	Advanced Cell Diagnostics	487751
Mm-Pirb	Advanced Cell Diagnostics	496031
Mm-Slc2a3	Advanced Cell Diagnostics	438851
Mm-Spry2	Advanced Cell Diagnostics	425061
Mm-Birc5	Advanced Cell Diagnostics	422701
Mm-Notch2	Advanced Cell Diagnostics	425161
Hs-TUBA1B	Advanced Cell Diagnostics	529451
Mm-Myh9	Advanced Cell Diagnostics	556881
Mm-Nlrp3	Advanced Cell Diagnostics	439571
Mm-Cdkn1b	Advanced Cell Diagnostics	499991
Mm-Pla2g7	Advanced Cell Diagnostics	453811
Mm-Ppib	Advanced Cell Diagnostics	313911
Dap8	Advanced Cell Diagnostics	310043
**Reagents and Kits Critical for Immunohistochemistry and *in situ* hybridization**		
SCEM (embedding medium)	http://section-lab.jp/index.html	SCEM
Cryofilm type 2C (Adhesive film)	http://section-lab.jp/index.html	Cryofilm type 2C
**Critical Commercial Assays**	
**mRNA-Seq on C1**		
Nextera XTIndex Kit V2 setB	Illumina	15052164
TRuSeq Dual Index Sequencing Primers-Paired End	Illumina	15029399
Nextera XT Sample Prep Kit	Illumina	15032354
C1 Single-Cell Auto Prep Module2	Fluidigm	100–5519
Module2 (mRNA Seq)	Fluidigm	100–6209
Quant-iT PicoGreen dsDNA Assay Kit	Molecular Probes	P11496
Advantage 2 PCR kit	Takara-Clontech	639207
SMART-Seq v4 Ultra Low Input RNA Kit for the Fluidigm C1 System	Takara-Clontech	635028
SMARTer Ultra Low RNA Kit for the Fluidigm C1 System	Takara-Clontech	634835
DynaMag—PCR	Invitrogen	49–2025
Agencourt AMPure XP	Beckman-Coulter	A63880
LIVE/DEAD Viability/Cytotoxicity Kit	Invitrogen	L3224
Cell strainer, 40 μm	Falcon	352340
**qPCR on C1**		
SsoFast EvaGreen Supermix with low ROX	Bio-Rad	172–5211
DNA Suspension Buffer	Teknova	T0221
Single Cell-to-CT Kit	Invitrogen	4458237
GE 96.96 Dynamic Array DNA Binding Dye Loading Reagent Kit	Fluidigm	100–3415-R
**ddPCR on QX200^TM^ AutoDG^TM^ Droplet Digital^TM^ PCR System**		
ddPCR^TM^ 96-Well PCR Plates	Bio-Rad	12001925
DG32^TM^ Cartridges	Bio-Rad	1864108
PCR PlateHeat Seal, foil, pierceable	Bio-Rad	1814040
Automated Droplet Generation Oil for EvaGreen	Bio-Rad	1864112
ddPCR^TM^ Droplet Reader Oil	Bio-Rad	1863004
**Deposited Data**		
FACS-purified adult cochlear supporting cell single-cell RNA-Seq (Fluidigm C1)	This article	
**Experimental Models: Organisms/Strains**		
Tg(Lfng-EGFP)HM340Gsat BAC transgenic mouse line (Lfng^EGFP^)	GENSAT (Gong et al., 2003)	
**Software and Algorithms**		
Seurat v2.0	https://satijalab.org/seurat/	
SINGuLAR v3.6.2	https://www.fluidigm.com/software	

*Contact for Reagent and Resource Sharing.

### Experimental Model and Subject Details

#### Mice

CD-1 mice were obtained from Charles River Laboratories. CBA/J mice were obtained from Jackson Laboratories. The Tg(Lfng-EGFP)HM340Gsat BAC transgenic mouse line (*Lfng*^EGFP^) was generated by the GENSAT Project (Gong et al., 2003) and was kindly provided by A. Doetzlhofer (Johns Hopkins University). P60 and P120 mice of either sex were used for all experiments. Four mice were utilized for each experiment. All experiments were conducted in accordance with NIH animal use protocol 1379-15.

### Method Details

#### Adult Cochlea Preparation

Cochleae from P60 and P120 *Lfng*^EGFP^ mice were used for FACS purification of GFP+ SCs before single-cell capture. Apical to basal distribution of GFP+ SCs in the adult cochlea were determined by whole-mount and orthogonal immunohistochemistry (Supplementary Figures S1, S2). Briefly, the inner ear was dissected from adult mice and the vestibular portions of the inner ear, including utricle, saccule and semicircular canal ampulla, were removed. The apex of the cochlear bony labyrinth was exposed and an approximately 2 mm opening was made at the apex using fine forceps. This opening, along with the opening at the base of the cochlea that resulted from removing the vestibular portions of the inner ear allowed for good perfusion of fixative throughout the cochlea. The cochleae were then collected in a 1.5-ml tube (*n* = 8 cochleae per integrated fluidics chip (IFC) capture) and incubated in 0.05% crude trypsin (Worthington, Columbus, OH, USA) in CMF-PBS (Life Technologies, Carlsbad, CA, USA) at 37°C for 8 min. Excess trypsin solution was removed and four volumes of 5% FBS (Thermo Fisher Scientific, Waltham, MA, USA) in DMEM/F12 (Thermo Fisher Scientific, Waltham, MA, USA) was added to inactivate any remaining trypsin. The tissue was then triturated for 2 min and passed through a 40-μm strainer (pluriSelect Life Science, Leipzig, Germany) to eliminate residual aggregates and bone fragments. The resulting single-cell suspension was then stained with propidium iodide (Life Technologies, Carlsbad, CA, USA) to allow for the exclusion of dead cells and debris from the samples.

##### Flow Cytometry and Sample Collection

Single cells were sorted on a FACS Aria flow cytometer (BD Biosciences, San Jose, CA, USA) with a compensated FITC setting and 488 nm excitation, using a 100-μm nozzle. In adult tissue, GFP+ SCs are the brightest population and typically comprised 2–7% of viable cells (approximately 10,000–35,000 GFP+ SCs from 500,000 total cells from eight cochleae). The purpose of FACS purification was to enrich for a population of GFP+ SCs and gating was set based on wild type isotype controls to exclude the majority of GFP– cochlear cells (see example of FACS gating on Supplementary Figure S3). Scatter discrimination was used to eliminate doublets and samples were negatively selected with propidium iodide to remove dead cells. Cells were collected in 20% FBS in DMEM/F12 and stored on ice. After sorting, cells were spun at 200 g for 10 min and then resuspended in 20% FBS. More detailed descriptions of the isolation methods have been previously published (Burns et al., 2015).

##### Adult Cochlear Single Supporting Cell RNA-Seq Using the Fluidigm C1 Platform

Methodology for single-cell RNA-Seq on the Fluidigm C1 Platform was previously described (Burns et al., 2015). Briefly, cell capture, lysis, SMARTer-based RT and polymerase chain reaction (PCR) amplification of cDNA was performed as outlined in the Fluidigm protocol (PN 100–5950 B1). After obtaining a single-cell suspension, 10 μl of cells at a final concentration of 2.5 × 10^5–^7 × 10^5^ cells per μl were loaded onto a medium-sized (10–17 μm) IFC. Cell concentration was estimated at a 1:10 dilution using an automated cell counter (Luna). The IFC was placed in the C1 system, where cells were automatically washed and captured. After capture, the chip was removed from the C1 and a 30-μm stack of widefield fluorescence and brightfield images was recorded at each capture site using an ×10/0.4 numerical aperture objective on an inverted Zeiss Axio Observer.Z1 microscope equipped with a motorized stage (example image in Supplementary Figure S3). Automated imaging was performed using a custom script written in the Zeiss Zen Blue software as described previously (Burns et al., 2015). Average imaging time for all 96 capture sites was 35 min. A summary of each C1 capture can be found in Supplementary Table S1. After the imaging period, the IFC was returned to the C1 where lysis, RT, and PCR were performed automatically within the individual reach chambers for each cell. For RNA-Seq, mixes were prepared from the SMARTer Ultra Low RNA kit (Clontech) according to the volumes indicated in the Fluidigm protocol. For qPCR, mixes were prepared from the Single Cell-to-CT qRT-PCR kit (Ambion, Austin, TX, USA). The thermal cycler within the C1 performs 21 or 18 rounds of PCR amplification to obtain enough material for RNA-Seq or qPCR, respectively. cDNA was manually collected from the output channel of each capture site and stored in a 96-well plate at –20°C until library preparation. The average time from dissection to cell lysis was approximately 4 h for FACS-purified cochlear SCs.

##### Single-Cell RNA-Seq Library Sequencing, Alignment, and Estimation of Gene Expression

Each collection of 96 pooled single-cell libraries was sequenced on a single flow cell lane of an Illumina HiSeq 1500 to an average depth of 1.8M reads using 90 × 90 paired-end reads. A total of 279 single-cell libraries were generated from FACS-purified P60 and P120 Lfng^EGFP^-positive adult cochlear SCs. The quality and read depth requirements of the library preparation and sequencing protocols used here have been described in detail elsewhere, and our own analyses were all in agreement with these published reports (Burns et al., 2015).

Reads were de-multiplexed and then aligned to a Bowtie index genome based on the NCBI-annotated mouse transcriptome (comprising 48,714 genes in GRCm38.vM11 genome and corresponding GTF) using STARv2.5. The sequences and identifiers for EGFP were appended to the genome FASTA and the GTF prior to creating the index used for alignment. For each cell (library), absolute counts were calculated using STARv2.5 and using the TranscriptomeSAM parameter, relative transcript abundances were estimated from the aligned reads using RSEM v1.2.19 (default parameters; Li et al., 2010; Li and Dewey, 2011). RSEM estimates transcript abundance in units of transcript per million (TPM). The abundances reported here are at the gene level, which RSEM calculates by summing the estimated transcript abundances for each gene. Alignment, absolute counts, and abundance estimation were carried out on the NIH/Helix Biowulf cluster. Quality metrics were calculated using the CollectRNASeqMetrics in Picard (Supplemental data file: multiqc_report). Subsequent analysis has been performed with the dataset in absolute count format. Data in TPM format along with the raw data and quality metrics are provided for comparison to previously deposited FACS-purified P1 cochlear SCs (Burns et al., 2015; GEO Accession ID: GSE71982).

##### Outlier Identification

Cells that appeared unhealthy, as noted by lack of GFP or fragmented cellular appearance in the recorded capture site images, were excluded from library preparation. To further identify potentially unhealthy cells with abnormally low expression levels, we passed the cells through the outlier identification function provided in SINGuLAR Analysis Toolset 3.5.2, Fluidigm’s R package for single-cell expression analysis. Outlier identification in SINGuLAR proceeds by trimming low-expressing transcripts until 95% of the transcripts that remain are above 1 nTPM in half of the cells. A distribution of combined transcript expression is created from these cells, and outliers are considered as cells whose median expression across the identified gene list is below the 15th percentile of the distribution. Initial validation of clustering and close examination of whole-mount adult Lfng-EGFP mice cochleae revealed an additional population of Lfng^EGFP^-positive cells that constituted endothelial cells of capillaries in the spiral ganglion region. This along with the previously published outlier analysis methodology resulted in a final dataset of 211 adult cochlear SCs after the exclusion of outliers. Using these routines, 68 out of 279 adult cochlear SCs were excluded from the analysis (Supplementary Table S1).

##### PCA and *t*-SNE Analysis

###### Selection of Genes for Clustering Analysis

Identification of the highly variable genes was performed in Seurat utilizing the MeanVarPlot function and the default settings with the aim to identify the top ~2,000 variable genes (Satija et al., 2015). Briefly, to control for the relationship between variability and average expression, average expression and dispersion is calculated for each gene, placing the genes into bins, and then a *z*-score for dispersion within the bins was calculated. These genes are utilized in the downstream analyses for clustering.

###### Clustering of Single Cells

Clustering analyses of single-cell data was performed with Seurat using a graph-based clustering approach (Satija et al., 2015). Briefly, the Jackstraw function using the default settings was used to calculate significant principal components (*p* < 0.0001) and these principal components were utilized to calculate the k-nearest neighbors (KNN) graph based on the Euclidean distance in PCA space. The edge weights are refined between any two cells based on the shared overlap in their local neighborhoods (Jaccard distance). Cells are then clustered according to a smart local moving algorithm (SLM), which iteratively clusters cell groupings together with the goal to optimize the standard modularity function (Blondel et al., 2008; Waltman and van Eck, 2013,^1^). Resolution in the FindClusters function was set to 0.8. High modularity networks have dense connections between the nodes within a given module but sparse connections between nodes in different modules. Clusters were then visualized using the *t-distributed* stochastic neighbor embedding (t-SNE) plot.

###### Differential Gene Expression Analysis

Differential expression analysis was performed in Seurat utilizing the FindAllMarkers function with the default settings except that the “min.pct” and “thresh.use” parameters were utilized to identify broadly expressed (min.pct = 0.8, thresh.use = 0.01) and subpopulation-specific (min.pct = 0.5, thresh.use = 0.25) expression profiles. The parameter “min.pct” sets a minimum fraction of cells that the gene must be detected in all clusters. The parameter “thresh.use” limits testing to genes which show, on average, at least X-fold difference (log-scale) between groups of cells. The default test for differential gene expression is “bimodal,” a likelihood-ratio test (McDavid et al., 2013). Differentially expressed genes were then displayed on violin plots based on unbiased clustering described above. Default parameters for significance included a minimum of 25% of cells expressing a given gene and a fold change of at least 1.7.

###### Validation of qPCR Primer Set Assays

A total of 96 DELTAgene qPCR gene expression assays, consisting of forward and reverse qPCR primers, were purchased from Fluidigm. Ninty-three of the 96 assays were used for single-cell qPCR analysis in this study. DELTAgene assays were validated against adult mouse cochlea cDNA. To determine primer efficiencies for all 96 primer sets, we made eight 3-fold dilutions of the preamplification product and tested them in triplicate on a 96.96 Dynamic Array IFC using a Fluidigm BioMark HD microfluidics-based qPCR system. Primer efficiency was calculated with the formula: *E* = 10 (−1/slope), where slope represents the linear slope of a linear regression fit to the average standard curve. Primer sets with efficiencies ±0.2 from the ideal efficiency of 2, or primer sets where multiple peaks were detected on a melt curve were excluded from further analysis.

##### Validation of Adult Cochlear Supporting Cell Gene Expression With Single-Cell qPCR and Digital Droplet PCR (ddPCR)

###### Single-Cell qPCR

For single-cell qPCR, we captured single cells from P60 cochlea using medium-sized microfluidic chips (C1 Single-Cell Auto Prep IFC for PreAmp) as outlined in the Fluidigm protocol (PN 100–6117 G1). Mixes for lysis, RT, and specific target amplification were prepared from the Single Cell-to-Ct qRT-PCR kit (Ambion, Austin, TX, USA) and pre-designed Delta Gene assays (Fluidigm, South San Francisco, CA, USA). In addition to known markers, putative adult cochlear SC markers were selected for analysis in an arbitrary manner.

After 18 cycles of preamplification, expression levels using single-cell qPCR were performed on the Fluidigm Biomark HD system as previously described (Honda et al., 2017). cDNA from single cells was selected for qPCR in the same way as it was selected for RNA-Seq. A total of 170 single cells from four C1 captures were profiled using two Dynamic Array IFCs. Empty wells with primers were utilized for negative controls. The threshold of cycles (C_t_) values was calculated with Fluidigm Real-time PCR analysis software with the following settings: quality threshold of 0.65; a linear (derivative) baseline correction; and auto (detectors) method. We defined gene expression levels as log_2_ expression = LOD − C_t_, in which C_t_ = 24 was set as the LOD. We used the log_2_ expression dataset for hierarchical clustering. The SINGuLAR package v3.6.1 was utilized to display and analyze single-cell qPCR data.

###### Digital Droplet PCR (ddPCR)

FACS-purified adult cochlear SCs were collected from P60 Lfng^EGFP^ mice as detailed previously. RNA was isolated from 30,000 GFP-positive and GFP-negative cells with each assay standardized to 2,000 cells per sample collected in triplicates to use for the quantification experiment. The ddPCR Droplets were generated using the QX200 AutoDG Droplet Digital. PCR was performed as described in the QX200^TM^ ddPCR^TM^ EvaGreen^®^ Supermix instructions^2^. Droplets were read with a QX200^TM^ Droplet Reader (BioRad) and analyzed with QuantaSoft software (BioRad). Primer sequences utilized for ddPCR are provided in the Supplementary Table S3.

###### Co-localization With Immunofluorescence and Single-Molecule Fluorescent *in situ* Hybridization (smFISH)

For cochlear whole mounts, cochleae were fixed in fresh 4% paraformaldehyde in PBS overnight at 4°C. After fixation, specimens were washed in PBS then permeabilized and blocked for 1 h at room temperature in PBS with 0.2% Triton X-100 (PBS-T) with 10% fetal bovine serum. The samples were then incubated in the appropriate primary antibodies overnight at 4°C in PBS-T with 10% fetal bovine serum, followed by three rinses in PBS-T and labeling with AlexaFluor-conjugated secondary antibodies (1:250, Life Technologies, Carlsbad, CA, USA) in PBS-T for 1 h at room temperature. Where indicated, 4,6-diamidino-2-phenylindole (DAPI; 1:10,000, Life Technologies, Carlsbad, CA, USA) was included with the secondary antibodies to detect nuclei. Organs were rinsed in PBS three times and mounted in SlowFade (Invitrogen). Specimens were imaged using a Zeiss LSM 710 confocal microscope.

For immunohistochemistry and *in situ* hybridization of cochlear sections, fixed adult mouse inner ears were decalcified in 150 mM EDTA for 5–7 days, transferred to 30% sucrose, and then embedded and frozen in SCEM tissue embedding medium (Section-Lab Company Limited, Hiroshima, Japan). Adhesive film (Section-Lab Company Limited, Hiroshima, Japan) was fastened to the cut surface of the sample in order to support the section and cut slowly with a blade to obtain 6 μm thickness sections. The adhesive film with section attached was submerged in 100% EtOH for 60 s, then transferred to distilled water. The adhesive film consists of a thin plastic film and an adhesive and it prevents specimen shrinkage and detachment. This methodology allows for high-quality anatomic preservation of the specimen. The sections were cut to a thickness of 10 micrometers. Fluorescent immunohistochemistry was performed as described above. The sections were mounted with SCMM mounting medium (Section-Lab Company Limited, Hiroshima, Japan).

Fluorescent *in situ* hybridization was performed using RNAscope^®^ probes against candidate genes per previously published methodology and overlaid with fluorescent IHC (Wang et al., 2012). Briefly, tissue preparation was modified from the original published protocol (Advanced Cell Diagnostics, Newark, CA, USA, 320534) to avoid tissue detachment during the target retrieval step. Drying after sectioning was extended to 2 h followed by 1 h post-fixation in 4% PFA. A 30 min dry baking step at 60°C was added before protease treatment. Hybridization and detection were performed as described in RNAscope^®^ 2.5 HD Detection Reagent—RED User Manual Part 2 (Document Number 322360-USM) without hematoxylin counterstaining. The following RNAscope^®^ probes were used: human *TUBA1B* (Hs-TUBA1B), mouse *Myh9* (Mm-Myh9, 556881), mouse *Cdk1nb* (Mm-Cdkn1b, 499991), mouse *S100a6* (Mm-S100a6, 412981), mouse *Lcp1* (Mm-Lcp1, 487751), mouse *Notch2* (Mm-Notch2, 425161), mouse *Nlrp3* (Mm-Nlrp3, 439571), mouse *Slc2a3* (Mm-Slc2a3, 438851), mouse *Pla2g7* (Mm-Pla2g7, 453811), mouse *Spry2* (Mm-Spry2, 425061), mouse *Birc5* (Mm-Birc5, 422701), and mouse *Notch2* (Mm-Notch2, 425161). The target genes and probed regions are listed in Supplementary Table S4. Sequences of target probes, preamplifier, amplifier, and label probe are proprietary (Advanced Cell Diagnostics, Newark, CA, USA). For fluorescent detection, the label probe was conjugated to Alexa Fluor 555. All probes were purchased with conjugation from ACDBio. Assays were performed in parallel with positive (Ppib) and negative (dapB) controls.

For immunostaining following *in situ* hybridization, slides were washed 3 × 10 min in PBS-T and blocked for 3 h in 10% FBS before standard immunofluorescence procedure detailed previously (Burns et al., 2015). DAPI counterstaining was performed to label cell nuclei. The following primary antibodies were used: rabbit anti-Myosin VIIA (1:250; Proteus BioSciences, Ramona, CA, USA, 25-6791), sheep anti-S100a6 (1:100; R&D, AF4584), rabbit anti-Lcp1 (1:100; Cell Signaling, Danvers, MA, USA, 3588S), and mouse anti-Acetylated tubulin (1:250; Sigma–Aldrich, St. Louis, MO, USA). Candidate genes were tested on at least three adult mouse specimens from three different mice.

###### Human Temporal Bone Fluorescent Immunohistochemistry

Immunohistochemistry on human temporal bones was performed as previously described by Lopez et al. (2016). Detailed procedures of temporal bone collection, fixation, decalcification, and celloidin embedding were described by Merchant (Merchant, 2010). The methodology to mount celloidin-embedded sections, celloidin removal and antigen retrieval steps has been described in detail (Huang, 1975; Shi et al., 1998; O’Malley et al., 2009a,b) and used previously by the authors (Lopez et al., 2016). Antigen retrieval was performed as described previously (Lopez et al., 2016). Briefly, sections were heated in a microwave oven using intermittent heating methods of two 2-min cycles with an interval of 2 min between the heating cycles in 1:200 diluted antigen retrieval solution in water (Vector Antigen Unmasking Solution, Vector Labs, Burlingame, CA, USA). The petri dish containing the slides was removed from the microwave oven and allowed to cool for 15 min at room temperature and washed with PBS for 10 min prior to immunohistochemistry. Quenching of auto-fluorescence prior to immunohistochemistry was performed as described to remove auto-fluorescence intrinsic to the human temporal bone sections (Lopez et al., 2016). Briefly, sections were placed in a glass Petri dish containing ice-cold PBS and placed in a UV chamber for 8 h. Temperature was checked continuously to avoid overheating, and cold PBS was replaced every 30 min. The sections were processed for immunofluorescence once the auto-fluorescence signal in the tissue sections disappeared. Immunofluorescence was performed as previously described with hydrogen peroxide incubation step omitted. The sections were incubated with primary antibodies for 48 h at room temperature and secondary antibodies for 2 h at room temperature. The following primary antibodies were used: sheep anti-S100a6 (1:100; R&D, AF4584), rabbit anti-Lcp1 (1:100; Cell Signaling, Danvers, MA, USA, 3588S), mouse anti-Acetylated tubulin (1:250; Sigma–Aldrich, St. Louis, MO, USA), and calbindin (1:100; Santa Cruz, sc-365360). Candidate genes (sheep anti-S100a6, rabbit anti-Lcp1) were tested on at least three human specimens each from different individuals. Negative controls consisted of secondary antibody only and unstained human sections to assess for background staining and autofluorescence, respectively. Negative controls exhibited minimal staining or autofluorescence (data not shown).

###### Gene Ontology, Gene-Set Enrichment Analysis and Pathway-Enrichment Analysis of Predicted Proteins

Gene ontology analysis and gene enrichment analysis were performed using Enrichr^3^ as previously described (Chen et al., 2013; Kuleshov et al., 2016; Pazhouhandeh et al., 2017). Enrichr is an integrated web-based application that includes updated gene-set libraries, alternative approaches to ranking enriched terms, and a variety of interactive visualization approaches to display the enrichment results. Enrichr employs three approaches to compute enrichment as previously described (Jagannathan et al., 2017). The combined score approach where enrichment was calculated from the combination of the *p*-value computed using the Fisher exact test and the *z*-score was utilized. Graphically, the brighter the red and longer the bar, the greater the enrichment score based on the combined score approach for a given GO term. In order to visualize molecular interaction networks, the list of putative proteins inferred from genes expressed by adult cochlear SCs was introduced to STRING 10.0^4^ and the nodes (proteins) and edges (protein-protein interactions) were extracted (Szklarczyk et al., 2015). Proteins were linked in STRING based on the default medium (0.400) minimum interaction score and on the following seven criteria: text mining, experiments, databases, co-expression, neighborhood, gene-fusion, and co-occurrence. Interaction evidence from all utilized criteria are benchmarked and calibrated against previous knowledge, using the high-level functional groupings provided by the manually curated Kyoto Encyclopedia of Genes and Genomes (KEGG) pathway maps (Szklarczyk et al., 2015). The summation of this interaction evidence is utilized to construct a minimum interaction score.

### Quantification and Statistical Analysis

#### Experimental Design

For mouse experiments, all groups consisted of age-matched mice and n represents the number of independent biological replicates. Both male and female mice were pooled for all experiments. All data were included in the analysis and thus no exclusion criteria were used. Experiments were not blinded. Sample size estimates were not performed beforehand. Details are provided in the detailed “Materials and Methods” section.

#### Statistical Analysis

General statistical analysis for Figures (Bulk qPCR) was performed using *n* biological replicates as indicated in the detailed methods. Error bars indicated mean ± SEM, and statistical differences were assessed by a *t*-test. Statistical analysis for single-cell RNA-Seq is described in the detailed methods.

### Data and Software Availability

All data generated in these studies were deposited in the Gene Expression Omnibus (GEO) database (GEO accession ID: GSE135703). The dataset has been uploaded into the gene Expression Analysis Resource (gEAR), a website for visualization and comparative analysis of multi-omic data, with an emphasis on hearing research and the permalink for the dataset is: https://umgear.org/p?l=9f88d6fb.

## Results

### Adult Cochlear Supporting Cells Are Transcriptionally Distinct From Perinatal Cochlear Supporting Cells

**Figure 1:**
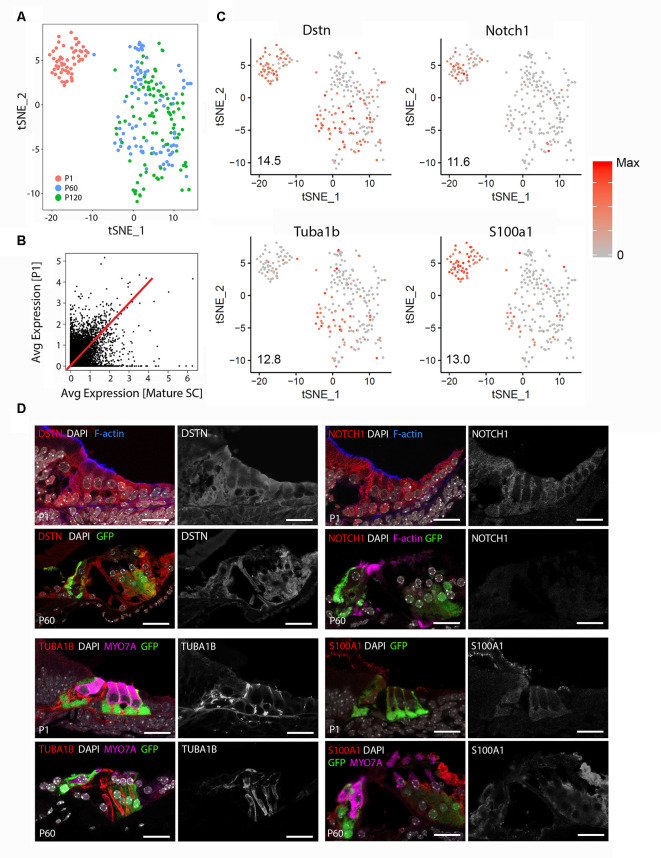
Adult cochlear supporting cells (SCs) are transcriptionally distinct from perinatal cochlear SCs. **(A)** Unbiased clustering of FACS-purified P1 and mature (P60, P120) cochlear SC transcriptomes demonstrates the clustering of single cells based on the transcriptional expression profiles for each cell. Note that P1 and mature cochlear SCs cluster within their respective groups but exhibit distinct clustering from each other. **(B)** Comparison of averaged gene expression between FACS-purified mature (P60, P120) and P1 cochlear SCs indicates both equivalent (genes expressed on or near the red line) and differential (genes located closer to either axis) expression between the two cell stages. **(C)** Feature plots of select known cochlear SC genes (*Dstn, Notch1, S100a1, Tuba1b*) demonstrate distinct differences between P1 and mature cochlear SCs. An expression is shown in log2 [nTPM] with the maximum expression value (Max) shown in the lower-left corner of each plot. The expression histogram is shown with red indicating higher expression. **(D)** Representative immunohistochemistry validating transcriptional differences between P1 and mature cochlear SCs in the organ of Corti from Lfng^EGFP^ mice. Each 4-panel grouping demonstrates P1 and P60 immunohistochemistry with the protein of interest in the red channel (left panels) at P1 (upper left panel) and P60 (lower left panel) and the gray scale single-channel images of the protein of interest (right panels) at P1 (upper right panel) and P60 (lower right panel). Known protein expression (DSTN, NOTCH1, S100A1, TUBA1B) is demonstrated (Upper left Four panels and proceeding clockwise). Staining for F-actin or MYO7A identifies hair cell (HC) stereocilia or HCs, respectively. Scale bar, 20 μm.

Unbiased clustering of the P1 and mature (P60 and P120) cochlear SC transcriptomes reveals that FACS-purified adult cochlear SCs demonstrate broadly distinct differences from previously published FACS-purified P1 cochlear SCs (Burns et al., 2015; Figure 1A). No differences in clustering or gene expression were observed between P60 and P120 cochlear SCs and for these reasons the two data sets are grouped together as mature SCs for the remainder of the analyses (data not shown). Supplementary Figure S4 demonstrates the degree of correlation in average gene expression as well as the large cell-to-cell variability in gene expression between P1 and mature cochlear SCs. The non-averaged gene expression data (Figure 1B; Supplementary Figure S4A) highlight the distinct gene expression profile of mature cochlear SCs. Using the Seurat FeaturePlot function, the differential distribution of four genes that have been reported to be expressed in adult SCs (*Dstn, Tuba1b, Notch1, S100a1*) were examined. Results are consistent with transcriptional changes in SCs between P1 and adult (Tannenbaum and Slepecky, 1997; Saha and Slepecky, 2000; Coppens et al., 2001; Murata et al., 2006; Oesterle and Campbell, 2009; Herde et al., 2010; Maass et al., 2015; Figure 1C). To confirm the results obtained from the single-cell analysis, immunohistochemical analysis for the four known marker genes was performed in P1 and P60 cochlear sections (Figure 1D). DSTN protein expression is present in both P1 and P60 cochlear SCs but appears less prominent and more confined to SCs at P60. NOTCH1 protein is expressed in cochlear SCs at P1 but is absent at P60. This observation is consistent with those reported by Murata et al. (2006) with NOTCH1 protein being present between P0 and P3 but absent at P7 in mouse cochlear SCs. For this reason, the observation of a few adult SCs expressing *Notch1* RNA likely represents stochastic expression. TUBA1B protein (acetylated tubulin antibody) expression is predominantly in the pillar cells at P1 with expression noted in the peripheral axons of the spiral ganglion neurons that make contact with the HCs. In contrast, SCs (pillar cells and Deiters cells) at P60 exhibit diffuse expression of TUBA1B protein. S100A1 protein is expressed in inner HCs and cochlear SCs at P1 but is restricted to inner border, inner phalangeal and the outermost Deiters cell in P60. These differences in transcriptome and accompanying protein expression support the transcriptional distinctiveness of mature cochlear SCs from their perinatal counterparts.

### An Overview of Adult Supporting Cell Single-Cell RNA-Seq

**Figure 2:**
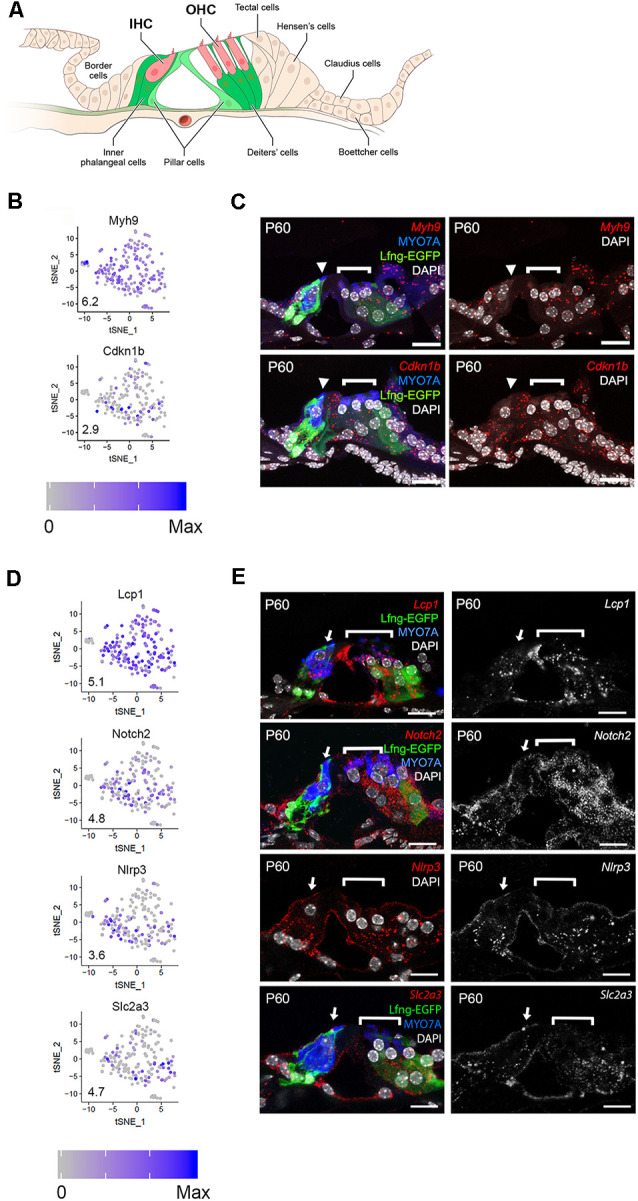
Single-cell RNA-Seq identifies adult SC gene expression. **(A)** Schematic of adult *Lfng^EGFP^* organ of Corti. GFP-expressing cells include inner phalangeal cells, Deiters’ cells, and, to a lesser extent, pillar cells. IHC = inner hair cell, OHC = outer hair cell. Mice that are homozygous and heterozygous for the transgene display the same phenotype. **(B)** Expression of genes that are known to be expressed by adult cochlear SCs (*Myh9, Cdkn1b*). Feature plots show the expression level of each gene in each cell. An expression is shown in log2 [nTPM] with the maximum expression value (Max) shown in the lower-left corner of each plot. The expression histogram is shown with blue indicating higher expression. **(C)** smFISH localization of RNA expression for *Myh9*, and *Cdkn1b* in cross-sections of the adult organ of Corti demonstrates localization in all SCs (border, inner phalangeal, the pillar, and Deiters cells). Upper left image demonstrates the *Myh9* probe in red along with Lfng-EGFP in green, antibody labeling forMYO7A (HCs), and DAPI-labeling of nuclei. Lower left image shows the *Cdkn1b* probe in red, Lfng-EGFP in green, MYO7A (HCs), and DAPI-labeling of nuclei. Upper and lower panels to the right illustrates the smFISH channel with DAPI-labeling of nuclei. **(D)** Expression of previously uncharacterized adult cochlear SC genes (*Lcp1, Notch2*, *Nlrp3*, *Slc2a3*) presented as in panel **(B)**. **(E)** smFISH localization of RNA expression for *Lcp1, Notch2*, *Nlrp3*, and *Slc2a3* in adult cross-sections of the organ of Corti. Images to the right demonstrate the RNA probe of interest in red along with anti-MYO7A (HCs) in blue, Lfng-EGFP in green and DAPI in white where applicable. Images to the right are the smFISH probe alone in grayscale. The IHC (arrowhead) and OHC regions (bracket) are indicated. *Myh9* = Myosin heavy chain 9 gene; *Cdkn1b* = Cyclin-dependent kinase inhibitor 1b gene; *Lcp1* = Lymphocyte cytosolic protein 1 gene; *Notch2* = Notch receptor 2 gene; *Nlrp3* = NLR family pyrin domain containing 3 gene; *Slc2a3* = Solute carrier family 2 member 3 gene; MYO7A = myosin 7A protein; DAPI = 4’, 6-diaminodino-2-phenylindole; Scale bars in all panels, 20 μm.

As a first step in examining the adult cochlear SC scRNA-Seq data set, expression of a subset of candidate genes was examined. A representative cross-sectional schematic of the adult *Lfng^EGFP^* mouse organ of Corti is shown in Figure 2A with GFP-expressing cells colored in green with the lighter green in pillar cells denoting less prominent GFP expression by comparison with other SC types. GFP expression, likely representing produrance of the transgene, was used to enrich for adult cochlear SC populations. As discussed, SCs were collected based on the expression of GFP. However, initial differential expression analysis of cell clusters and close examination of whole-mount adult *Lfng^EGFP^* cochleae revealed an additional population of EGFP^+^ cells that constituted endothelial cells of capillaries in the spiral ganglion region. As these cells do not constitute SCs, data from these cells were removed based on the expression of known endothelial transcripts, and the resulting data set was subsequently filtered following an outlier analysis (see “Materials and Methods” section). The final dataset included transcriptomic values from 211 adult cochlear SCs. We detected 17,243 different genes in the entire SC dataset that had an expression value greater than zero. With an arbitrary value of 10 counts as a cutoff for background level of expression, 14,908 different genes were considered to be expressed in the entire adult cochlear SC dataset No differences in clustering or gene expression were observed between P60 and P120 SCs (data not shown) and so data from the two time-points were combined.

To examine the quality of the data set, we selected six genes, two known (*Myh9*, *Cdkn1b*) and four previously unreported (*Lcp1*, *Notch2*, *Nlrp3*, *Slc2a3*), that showed expression within the single-cell RNA-Seq dataset, to validate using smFISH and/or immunohistochemistry. MYH9 and CDKN1B have been reported to be expressed in most adult SCs (Löwenheim et al., 1999; Mhatre et al., 2004). Consistent with that, we observed a relatively uniform expression of both genes amongst adult cochlear SCs (Figure 2B) and is consistent with immunolabeling (Supplementary Figure S5). smFISH analysis also showed comparable levels of expression across the adult cochlear SCs (Figure 2C). *Lcp1* and *Notch2* showed similar broad patterns of expression (Figure 2D). smFISH results for *Lcp1* and *Notch2* were largely consistent with the scRNA-Seq results in that expression was observed in all SCs (Figure 2E). *Nlrp3* and, to a lesser extent, *Slc2a3* showed expression that appeared to be confined to a smaller subset of adult SCs (Figure 2D). smFISH results for *Nlrp3* and *Slc2a3* were inconsistent with scRNA-Seq results in that expression was observed in all SCs (Figure 2E). These conflicting results for *Nlrp3* and *Slc2a3* between scRNA-Seq and smFISH suggest that detection bias may account for decreased detection in one of the SC clusters observed in scRNA-Seq. Detection bias or “dropout” refers to an event where a transcript is not detected in the sequencing data due to a failure to capture or amplify it (Haque et al., 2017). Overall, the validation of these markers in adult cochlear SCs points to the utility of this scRNA-Seq dataset for examining SC-specific transcriptomes. Feature plots of additional adult SC candidate genes are provided in the Supplementary Figures S6, S7.

### Adult Cochlear Supporting Cells Can Be Categorized Into Two Subpopulations

**Figure 3:**
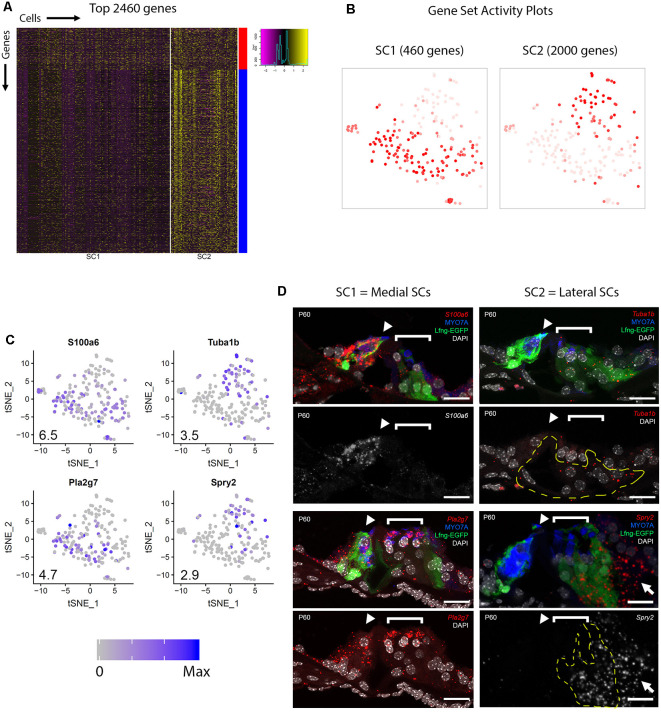
Adult cochlear SCs can be categorized into two subpopulations. **(A)** Heatmap depicting the top 2,460 genes expressed by the two clusters of adult cochlear SCs (SC1, SC2). For SC1, only 460 genes met criteria for significance, while for SC2, 2,000 genes that met criteria for significance were identified (see “Materials and Methods” section). Cells are arrayed along the horizontal axis and genes are arrayed along the vertical axis. Two main clusters of genes are distinguished by the color bars on the vertical axis to the right of the heatmap with the red bar corresponding to SC2 and the blue bar corresponding to SC2. **(B)** Gene set activity plots to demonstrate composite gene expression projected onto feature plots in SC1-defining (red bar, 460 genes) and SC2-defining (blue bar, 2,000 genes). Despite the visually apparent presence of some SC1-defining genes in the SC2 cluster of SCs, SC1-defining genes as a composite appear to define the SC1 adult cochlear SC subpopulation. **(C)** Feature plots of 1 known (*Tuba1b*) and 3 previously uncharacterized (*S100a6*, *Pla2g7*, *Spry2*) genes show differential expression between SC1 and SC2 SC subpopulations. *S100a6* and *Pla2g7* correspond to SC1 (red gene cluster in panel **A**) and *Tuba1b* and *Spry2* correspond to SC2 (blue gene cluster in **A**). Numbers in the bottom left corner of the feature plots represent maximum expression level (Max) among adult cochlear SCs. The expression histogram is shown with blue indicating higher expression. **(D)** smFISH localization of RNA expression for these four candidate genes. Below each color image with the RNA probe color designated in italics is either a grayscale single channel of the smFISH RNA probe or the smFISH RNA probe channel with DAPI-labeling of nuclei. *S100a6* and *Pla2g7* demonstrate higher levels of transcript expression in the medial SCs (inner border, inner phalangeal cells) while *Spry2* demonstrates higher levels of transcripts in the lateral SCs (predominantly Deiters cells as denoted by yellow-dashed line outline but also noted in Hensen’s cells as denoted by white arrow). Expression of Tuba1b transcripts can be seen in lateral SCs (pillar and Deiters cells) in the top image with Lfng-EGFP in green, MYO7A in blue (HCs) and DAPI-labeling of nuclei in the top right image for the *Tuba1b* RNA probe. The region of the lateral SCs (pillar and Deiters cells) is identified by the dashed yellow line in the Tuba1b smFISH probe image with DAPI-labeling of nuclei. HCs are labeled with MYO7A (blue), cell nuclei are labeled with DAPI (white) and Lfng-EGFP transgene expression (green) in SCs. The IHC (arrowheads) and OHC regions (bracket) are indicated. *S100a6* = S100 calcium-binding protein a6 gene; *Pla2g7* = Phospholipase A2 Group VII gene; *Tuba1b* = Tubulin alpha 1b gene; *Spry2* = Sprouty RTK signaling antagonist 2; MYO7A = myosin 7A protein; DAPI = 4’,6-diaminodino-2-phenylindole; Scale bar in all panels, 20 μm.

In order to gain a better overall perspective on the transcriptional diversity of adult cochlear SCs, the examination of a larger group of differentially expressed genes was performed. Unbiased clustering revealed two clusters of SCs, SC1 and SC2, as depicted in the heatmap (Figure 3A). Broadly comparing the two SC clusters, we detected 16,137 and 16,737 genes that had expression values greater than zero in SC1 and SC2, respectively. With an arbitrary value of 10 counts as a cutoff for background level expression, 10,188 and 13,613 genes were considered to be expressed in SC1 and SC2 cells, respectively, with 9,342 genes expressed in both populations. Two main clusters of genes are distinguished by the color bars on the vertical axis to the right of the heatmap with the red bar delineating 460 transcripts that demonstrate higher RNA expression in SC1 and the blue bar delineating 2,000 transcripts that exhibit higher RNA expression in SC2 (Figure 3A). A composite expression plot of the SC1- and SC2-defining profiles demonstrates that these transcripts appear to define two separate clusters of adult cochlear SCs, respectively (Figure 3B).

To examine whether the SC1 and SC2 might represent spatially distinct SC types, expression of four genes, including one known (*Tuba1b*) and three previously unreported (*S100a6*, *Spry2*, *Pla2g7*) genes, which showed differential expression between SC1 and SC2 (Figure 3C) were examined in cross-sections. TUBA1B has previously been shown to be expressed in the adult pillar and Deiters cells (Oesterle and Campbell, 2009). Our scRNAseq data supports this observation with significant differential expression between the SC1 (low) and SC2 (high; Figure 3C). Furthermore, the data is supported by immunolabeling for acetylated tubulin protein (TUBA1B) in Figure 1D as well as the expression of *Tuba1b* transcripts in lateral SCs (pillar and Deiters cells) demarcated by the yellow dashed lines compared to their absence in medial SCs (border and inner phalangeal cells) in the image for the *Tuba1b* RNA probe with only the nuclei labeled with DAPI (Figure 3D). In contrast with *Tuba1b*, *S100a6* showed higher expression in SC1 relative to SC2 (Figure 3C). Consistent with this result, smFISH indicated high levels of *S100a6* transcripts in medial SCs (border and inner phalangeal cells) and essentially no transcripts in lateral SCs (Figure 3D). *Spry2* showed higher expression in SC2 relative to SC1 while *Pla2g7* showed higher expression in SC1 relative to SC2 (Figure 3C). Consistent with these results, smFISH indicated high levels of *Spry2* transcripts in lateral SCs (predominantly Deiters cells demarcated by the yellow dashed lines) and higher levels of *Pla2g7* transcripts in the medial SCs (border and inner phalangeal cells) relative to the lateral SCs (pillar and Deiters cells; Figure 3D). Note the slightly higher intensity of *Spry2* RNA transcripts in Hensen cells (white arrow) compared to Deiters cells. Overall, these results confirm the expression of a subset of candidate genes in SCs and strongly suggest that SC1 and SC2 SC subpopulations, represent medial and lateral SCs, respectively. A description of these candidate adult supporting cell genes whose transcripts are validated in adult cochlear supporting cells is noted in Supplementary Table S5.

### Digital Droplet PCR (ddPCR) and Single-Cell qPCR Analyses of Additional Transcripts Validate Adult Cochlear Supporting Cell scRNA-Seq

**Figure 4:**
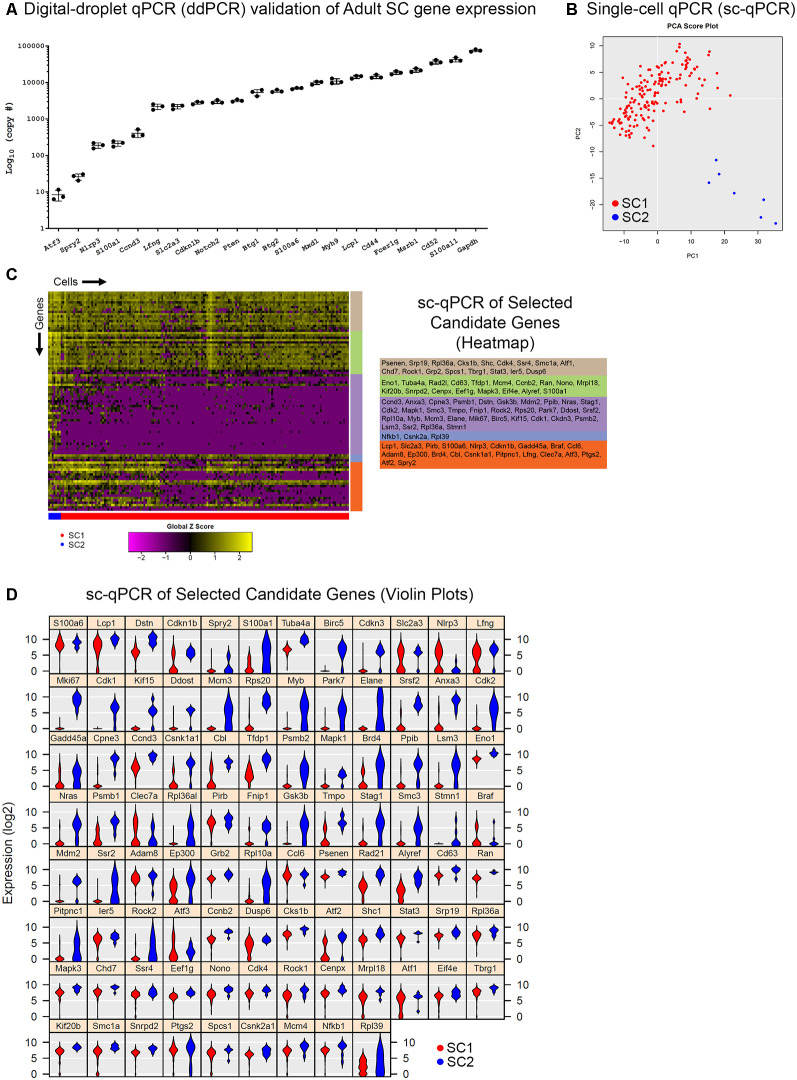
Digital droplet PCR (ddPCR) and single-cell quantitative polymerase chain reaction (qPCR) analyses of additional transcripts validate adult cochlear SC scRNA-Seq. **(A)** Digital droplet PCR (ddPCR) quantification of candidate genes in FACS-purified P60 *Lfng^EGFP^*-positive cochlear SCs. Absolute quantitation measured in a number of transcript copies detected are plotted on the log base 10 scale on the vertical axis and genes of interest are on the horizontal axis. All candidate genes were detected indicating expression in FACS-purified adult cochlear SCs. **(B)** Principal component analysis (PCA) of single-cell qPCR (sc-qPCR) of 170 FACS-purified P60 Lfng^EGFP^-positive cochlear SCs corroborates unbiased clustering of single-cell transcriptomes into two clusters (SC1 in red, SC2 in blue). **(C)** Heatmap of sc-qPCR results with 170 adult cochlear SCs (along the horizontal axis) and 93 candidate genes (along the vertical axis). Candidate gene clusters as determined by hierarchical clustering are noted as the colored bars along the vertical axis of the heatmap. Candidate genes making up the gene clusters are noted to the right of the heatmap in the corresponding colored boxes. As denoted by the expression histogram, the higher the expression, the more yellow the box corresponding to the gene in a given cell. **(D)** Violin plot display of sc-qPCR results demonstrates candidate gene expression levels in adult cochlear SCs.

While validation using smFISH and/or immunohistochemistry provides valuable spatial information regarding transcriptional expression, these methods are labor-intensive and low throughput. In our case, two cochlear SC subpopulations were identified in the data without a readily available method to specifically isolate these subpopulations from the adult mice. Therefore, we wanted to determine whether digital droplet PCR (ddPCR) and single-cell qPCR (sc-qPCR) could be used in combination as higher throughput methods for validating scRNA-Seq data. Specifically, the presence or absence of the genes of interest in FACS-purified GFP-positive adult cochlear SCs could be determined by ddPCR and the differential expression between two SC subpopulations could be confirmed with sc-qPCR. For these reasons, we identified an additional group of 93 differentially expressed genes between SC1 and SC2 cochlear SC subpopulations that were selected from the top 800 differentially expressed genes. Some genes were chosen due to preexisting knowledge about cochlear SCs (Tannenbaum and Slepecky, 1997; Saha and Slepecky, 2000; Malgrange et al., 2003; Shim et al., 2005; White et al., 2006; Oesterle and Campbell, 2009; Herde et al., 2010) while the remainder of the candidate genes were chosen at random. Using a subset of these genes, we confirmed the presence of their transcripts in populations of FACS-purified adult cochlear SCs using ddPCR where presence was measured in a number of copies per 2,000 cells (Figure 4A). Expression was present and quantifiable in each of the genes from this selected group. In order to validate the differential expression of genes between the two cochlear subpopulations (SC1 and SC2), we performed sc-qPCR on 170 FACS-purified GFP-positive cochlear SCs from the Lfng^EGFP^ adult mouse cochlea using this additional group of differentially expressed genes. Principal component analysis (PCA) corroborates unbiased clustering of single-cell transcriptomes into two clusters on the basis of these genes (Figure 4B). The expression of these genes is depicted in the heatmap (Figure 4C). Violin plots show the expression level of each of these genes as determined by sc-qPCR (Figure 4D). These data demonstrate the potential utility of a combinatorial approach to scRNA-Seq validation utilizing ddPCR to screen for the presence or absence of transcripts in a mixed population and then utilizing sc-qPCR to validate differential expression observations.

### Preliminary Evidence for Supporting Cell-Specific Genes Expression in Human Temporal Bones

To provide preliminary support for the relevance of SC markers identified in mice to human SCs, we localized the expression of two previously uncharacterized SC markers, S100A6 and LCP1, in organs of Corti from human temporal bone specimens and have provided supporting images (Supplementary Figure S8) along with explanatory text (Supplemental Datasheet S1) and references (Greenbaum et al., 2003; Jurewicz et al., 2018; Liu et al., 2018) as well as control images (Supplementary Figures S9, S10).

### Cell Cycle Phase Analysis Reveals That the SC2 Adult Supporting Cell Subpopulation Expresses Transcripts Associated With S Phase and G2/M Phase Canonical Markers

**Figure 5:**
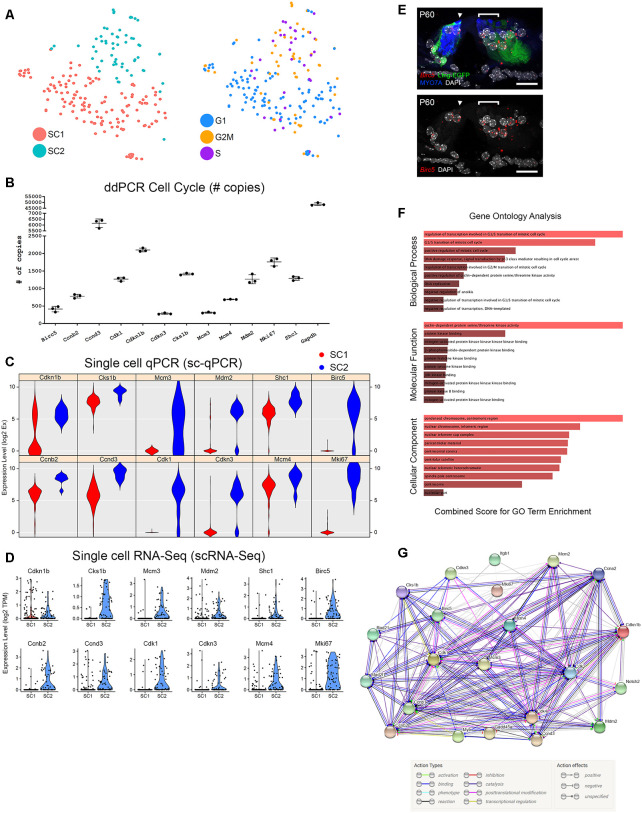
Cell cycle analysis of adult cochlear SCs. **(A)** Analysis of cell cycle phase among FACS-purified adult cochlear SCs reveals that the SC2 cluster of adult cochlear SCs express predominantly S phase and G2/M phase canonical markers compared to SC1 adult cochlear SC cluster. tSNE plot with the clustered cell types is shown to the left with an accompanying tSNE plot demonstrating cells clustered by cell cycle phase to the right. **(B)** Single-cell RNA-Seq expression of G2/M and S phase-specific markers. Violin plots for a select group of G2/M and S phase-specific cell cycle-related genes demonstrate predominant expression in the SC2 cluster of adult cochlear SCs. For violin plots, expression level (log2 TPM) is displayed on the vertical axis and cell cluster is displayed on the horizontal axis. **(C)** Single-cell qPCR of FACS-purified adult cochlear SCs for G2/M and S phase cell cycle-related markers confirms gene expression in adult cochlear SCs. Violin plots are redisplayed from Figure 4D for ease of comparison to the scRNA-Seq results above. **(D)** Digital droplet PCR (ddPCR) quantifies the presence of the select group of cell cycle-related genes validated by sc-qPCR in FACS-purified P60 Lfng^EGFP^-positive cochlear SC populations. **(E)** Cell cycle gene expression is demonstrated in mid-modiolar sections of the P60 mouse organ of Corti. RNA probe (in red) with accompanying immunohistochemistry is shown in image to the left and grayscale single-channel image is shown in image to the right. smFISH probe for *Birc5* (red dots) is shown to overlap with adult cochlear SCs (GFP in green). HCs are labeled with MYO7A (blue) and nuclei are labeled with DAPI (white). Location of IHC (arrowhead) and region of outer HCs (bracket) are denoted. Scale bar in all panels, 20 μm. **(F)** Gene Ontology (GO) analysis of cell cycle-related genes expressed by FACS-purified GFP-positive cells from P60 Lfng^EGFP^ cochlea suggests that these cells may be maintained in a non-proliferative state by a repressive network of genes. All cell cycle genes expressed by adult SCs from the dataset, regardless of which cluster of adult cochlear SCs expressed these genes, were used as the starting input in Enrichr. GO biological process analysis suggests that genes involved in the G1/S transition of the mitotic cell cycle are prominent in adult cochlear SCs. GO molecular function and cellular component analysis point to cyclin-dependent protein serine/threonine kinase activity and cellular components associated with condensed chromatin at the centromere, respectively. The color of the bar corresponds to the combined score which is calculated by taking the log value of the *p*-value from the Fisher exact test and multiplying this value by the *z*-score of the deviation from the expected rank. The longer and lighter colored bars indicate that the term is more significant. **(G)** Use of the STRING database to perform protein-protein interaction analysis identifies a set of interactions that may be related to the persistence of the post-mitotic state in adult cochlear SCs. The STRING plot demonstrates the action types and action effects as noted in the accompanying legend.

Focusing on mechanisms that might potentially facilitate mitotic regeneration in post-mitotic adult cochlear SCs, we utilized a recently-developed methodology to computationally assign cell cycle phase (G0/G1, S, or G2/M) based on cell cycle-specific gene expression to single cells on the basis of their single-cell transcriptomes (Scialdone et al., 2015; Nestorowa et al., 2016,^5^). PCA analysis suggests that the SC2 SC subpopulation which likely represents the lateral SCs (pillar and Deiters cells; Figure 3D) express transcripts that are consistent with S phase or G2/M phase while SC1 cochlear SCs do not (Figures 5A,B and Supplementary Figures S11, S12). Single-cell qPCR in Figure 5C validates these findings with select S phase (*Mcm4*) and G2/M (*Birc5, Cdk1, Mki67*) phase transcripts showing either predominant expression (*Birc5*, *Cdk1*, *Mki67*) or mild enrichment in transcript expression (*Mcm4*) in SC2 (Figure 5C). These data validate baseline expression levels of these selected S phases- and G2/M phase-associated transcripts. The validation lends support to the idea that the scRNA-Seq data could be utilized to assess baseline expression levels of S phase- and G2/M phase-associated genes, which could be modulated in the future in order to facilitate mitotic regeneration.

### Adult Cochlear Supporting Cells Continue to Express Some Cell Cycle Genes Expressed by Neonatal Lgr5+ Inner Ear Progenitors

One of the reasons for the interest in adult SCs is the thought that they might serve as a potential stem cell reservoir for regenerating HCs in the organ of Corti. Lgr5+ neonatal cochlear cells are thought to be potential progenitors for HCs and a potential stem cell reservoir within the organ of Corti (Shi et al., 2013; Wang et al., 2015; McLean et al., 2016; Cheng et al., 2017). For these reasons, similarities between Lgr5+ neonatal cochlear SCs and adult cochlear SCs gene expression were examined. Cheng et al. (2017) surveyed genes expressed by Lgr5+ neonatal cochlear SCs and reported expression of genes implicated in the cell cycle, Notch, EGF and Wnt signaling pathways. To examine whether some of these same genes might continue to be expressed in adult SCs, and in particular in the SC2 group, violin plots for a subset of these cell cycle genes were generated (Supplementary Figure S13). Using ddPCR, we confirmed the presence of the transcripts for a selected group of these cell cycle genes in FACS-purified GFP-positive adult cochlear SCs (Figure 5B). Differential expression of these cell cycle genes was then validated by sc-qPCR (Figures 4D, 5C). The results from the sc-qPCR from Figure 4D are redisplayed in Figure 5C to show a correlation between scRNA-Seq violin plots (Figure 5D) and the sc-qPCR violin plots (Figure 5C). Transcripts identified as being enriched in Lgr5-positive neonatal SCs and enriched in adult cochlear SCs by both scRNA-Seq and sc-qPCR include *Cdkn1b, Cks1b, Mcm3, Mdm2, and Shc3*. Transcripts identified as being enriched in Lgr5-negative neonatal SCs and enriched in adult cochlea SCs by both scRNA-Seq and sc-qPCR include *Birc5*, *Ccnb2*, *Ccnd3*, *Cdk1*, *Cdkn3*, *Mcm4*, and *Mki67*. *Cdkn1b, Mcm3*, *Mdm2*, *Birc5*, *Cdk1*, *Cdkn3*, and *Mki67* exhibit relatively good agreement between SC clusters in both scRNA-Seq and sc-qPCR. However, transcript expression, as determined by sc-qPCR, for *Cks1b, Shc1, Ccnb2, Ccnd3*, and *Mcm4* appears to contradict the results of scRNA-Seq in that expression is noted in the SC1 SC cluster. Reasons for this discrepancy between scRNA-Seq and sc-qPCR include the likelihood that PCR may be more sensitive at detecting transcripts given its targeted nature while scRNA-Seq must contend with potential inefficiencies in reverse transcription and amplification (Livak et al., 2013). smFISH validation of select cell cycle genes identified by Nestorowa et al. (2016) and Cheng et al. (2017) (*Notch2*, *Birc5*) is also performed in adult mouse organ of Corti and their mRNA expression overlaps with adult GFP-positive cochlear SCs (Figures 2F, 5E; Nestorowa et al., 2016; Cheng et al., 2017). A description of these candidate cell cycle genes whose transcripts are validated in adult cochlear SCs is noted in Supplementary Table S6. Shared gene expression of adult cochlear SCs with Lgr5+ neonatal cochlear SCs suggests that adult SCs may still possess latent abilities to serve as a stem cell reservoir.

### Adult Cochlear Supporting Cells May Be Maintained in a Non-proliferative State

Finally, to examine other pathways that are active in adult SCs, a gene ontology biological process analysis was performed. Results suggest that genes involved in the G1/S transition of the mitotic cell cycle are more prominently expressed in adult cochlear SCs by comparison with Lgr5+ neonatal cochlear SCs (Figure 5F). These genes may function to prevent adult SCs from re-entering or moving forward successfully through the phases of the cell cycle. In addition, gene ontology molecular function and cellular component analyses group these cell cycle genes to cyclin-dependent protein serine/threonine kinase activity and cellular components associated with condensed chromatin at the centromere, respectively, suggesting that these pathways could be activated in adult cochlear SCs and contribute to the maintenance of quiescence (Figure 5F). Use of the STRING database to perform protein-protein interaction analysis identifies a set of interactions that may be related to the persistence of the post-mitotic state in adult cochlear SCs (Figure 5G). Overall, these analyses suggest that these data could be utilized as a resource to explore mechanisms related to the maintenance of SC quiescence.

## Discussion

One of the major causes of sensorineural hearing loss in adults, a significant and irreversible health problem, is the loss of sensory HCs. While adult cochlear SCs may represent a potential source of replacement HCs, the hearing restoration will likely require regenerating the proper complement of cell types including any SCs that are converted to HCs and restoration of the architecture of the organ of Corti (Jahan et al., 2015). The exact temporal expression sequence and level of gene expression required for the creation of both mature HCs and SCs are yet to be elucidated. Furthermore, the adult organ of Corti appears to contain barriers to regeneration that remain incompletely defined. Improved understanding of the final mature state of cell types in the organ of Corti may contribute to future attempts at hearing restoration through regenerative approaches. These data provide gene expression levels for two groups of cochlear SCs that are targets for the induction of regenerated HCs. Many of the genes we have identified in these SCs have not been previously characterized in the inner ear and may be examined as possible targets for the development of SC-specific gene expression or as candidates for induction of changes in cell fate or mitotic state (Supplementary Table S2).

### Adult Cochlear Supporting Cells Are Transcriptionally Distinct From Perinatal Cochlear Supporting Cells

While previous studies have focused on identifying the genetic cascade necessary to convert perinatal cochlear SCs into HCs (Kelly et al., 2012; Liu et al., 2012; Costa et al., 2015; Walters et al., 2017), it seems possible that changes in the transcriptome and/or epigenetic state of adult SCs may necessitate activation of different pathways. Consistent with this hypothesis, the results presented here demonstrate that the transcriptomes of adult cochlear SCs are markedly different from P1 cochlear SCs (Burns et al., 2015; GEO Accession ID: GSE71982; Figure 1). Biological process gene ontology analysis reveals that P1 cochlear SCs demonstrate enrichment for genes involved in positive regulation of Wnt signaling (GO:0090263, GO:0030177), regulation of stem cell differentiation (GO:2000736), and mitotic cell cycle phase transition (GO:1901990), all of which could play a role in a regenerative response. In contrast, adult cochlear SCs demonstrate enrichment for genes involved in regulation of MAP kinase activity (GO:0043406, GO:0043405, GO:0000187), regulation of JNK cascade (GO:0046328), activation of protein kinase activity (GO:0032147), and integrin-mediated signaling pathway (GO:0007229). Work by others has suggested that modulation of MAP kinase signaling, JNK signaling, and protein kinase signaling may facilitate S-phase entry and proliferation (Montcouquiol and Corwin, 2001). These authors suggest that a balance between MAP kinase signaling and JNK signaling may determine whether cells proliferate or die by apoptosis. Work in zebrafish demonstrates that inhibition of JNK signaling leads to suppression of HC regeneration apparently by preventing proliferation, suggesting that JNK signaling plays a role in proliferation in inner ear sensory epithelia (He et al., 2016). However, Montcouquiol and Corwin (2001) suggest that activation of these signaling pathways (MAP kinase, JNK, protein kinase activity) may not be sufficient for proliferation. Davies et al. (2007) have also suggested that changes in the cell-extracellular matrix interactions as cells mature in the cochlea, notably changes in integrin expression, may maintain these cells in their postmitotic quiescent state. These observations combined with the absence of enrichment for genes involved in Wnt signaling and mitotic cell cycle transition may highlight some key differences between adult and perinatal cochlear SCs. Overall, these observations point to a need to better understand what distinguishes adult cochlear SCs from perinatal cochlear SCs (White et al., 2006). While it may seem obvious that adult cochlear SCs are transcriptionally distinct, much of the current work into elucidating the developmental transitions necessary for the production of new HCs and SCs presumes that these same developmental transitions can be applied to adult cochlear SCs *in vivo*.

### Single-Cell RNA-Seq Identifies Adult Supporting Cell Gene Expression

In order to demonstrate the utility of this dataset, we utilized smFISH to both confirm and localize transcript expression to adult cochlear SCs. smFISH validation of both known (*Myh9, Cdkn1b*) and novel (*Lcp1, Notch2*) genes shows relatively good concordance with scRNA-Seq data. While scRNA-Seq results for *Nlrp3* and *Slc2a3* show higher expression in SC1 compared to SC2, smFISH demonstrates presence across all SCs. Reasons for discrepancies between scRNA-Seq and sc-qPCR include the likelihood that PCR may be more sensitive at detecting transcripts given its targeted nature, while scRNA-Seq can show decreased sensitivity for particular transcripts because of inefficiencies in reverse transcription and amplification (Livak et al., 2013).

### Adult Cochlear Supporting Cells Can Be Categorized Into Two Subpopulations

Our data identified two transcriptionally distinct subgroups of adult SCs (SC1 and SC2). smFISH localization of *S100a6* and *Pla2g7*, markers of SC1, was largely restricted to medial SCs (border and inner phalangeal cells), while *Tuba1b* and *Spry2*, markers of SC2, were localized to lateral SCs (pillar and Deiters cells). More importantly, the composite expression of each of these two identified clusters of genes define these two groups as seen on the gene set activity plots (Figure 3B) and support the potential relevance of combinatorial gene expression in identifying subpopulations (Patel et al., 2014). These results are consistent with single-cell analysis of the developing cochlear duct which suggest that the organ of Corti is derived from two transcriptionally distinct regions, medial and lateral, with medial giving rise to inner HCs and associated SCs and lateral giving rise to outer HCs and associated SCs (Kolla and Kelley, personal communication). Since these regional distinctions could also be indicative of lineage restrictions, the identification of markers that define each domain could be utilized to create gene therapy targeting vectors specific for medial or lateral SC types (Stone and Cotanche, 2007; Cox et al., 2014). Comparison to existing adult SC (Liu W. J. et al., 2018; Ranum et al., 2019) and HC (Liu H. et al., 2014; Li Y. et al., 2018; Ranum et al., 2019) datasets has been provided as Supplementary Figures S14–S24 (Supplemental Datasheet S2).

### Population qPCR and Single-Cell qPCR Validation of Additional Gene Candidates From Adult Single-Cell Transcriptomes

Recently, single-cell RNA-sequencing through commercial droplet-based platforms has achieved higher throughput in comparison to microfluidics-based platforms, such as the Fluidigm C1 platform utilized in this study. However, because the Fluidigm system does not rely on molecular barcodes to discriminate cell of origin, ddPCR and single-cell qPCR can be used to examine the expression of specific transcripts within each data set, with a higher level of sensitivity by comparison with scRNA-Seq (Figures 4, 5). The results of our analysis of SCs using ddPCR and/or sc-qPCR yielded results that were largely consistent with the scRNA-Seq results (Figures 4, 5, Supplementary Figure S25). However, some differences were also observed. For instance, while *S100a6* transcript expression is increased in SC1 vs. SC2, a result that was observed by smFISH as well, single-cell qPCR (sc-qPCR) for *S100a6* demonstrates relatively equivalent levels of gene expression. This result could be a result of sampling bias, as only seven SC2 cells were collected for the sc-qPCR data set or might reflect decreased amplification efficiency for this primer set. Future efforts to increase the number of SC2 cells may address some of the discrepancies between the different methods.

### A Potential Role for Cell Cycle Regulation in Adult Cochlear Supporting Cells

Pathway analysis for the SC2 subpopulation indicated the expression of canonical S and G2/M phase genes and similarities to Lgr5-expressing cells isolated from the neonatal cochlea (Nestorowa et al., 2016; Cheng et al., 2017). These Lgr5-expressing cells in the neonatal cochlea possess a latent potential to proliferate and to convert into HCs (Stone and Cotanche, 2007; Cox et al., 2014). Mitotic regeneration has been explored in SCs as a potential avenue for HC regeneration with Wnt activation and Notch signaling playing roles in SC conversion into HCs (Ni et al., 2016; McGovern et al., 2018, 2019). Potentially consistent with this are protein interaction analyses that suggest interactions between Notch signaling and Wnt signaling pathways and cell cycle control, specifically *Cdkn1b* (Figure 5G). Work by others suggests that these signaling pathways may be relevant to achieving HC regeneration in the adult inner ear (Hori et al., 2007; Kuo et al., 2015; Walters et al., 2017; Liu H. et al., 2018).

While the overall biological process gene ontology analysis suggests involvement in the G1/S transition of the mitotic cell cycle, sub-analyses categorize these genes into three main categories: positive regulation of transcription from RNA polymerase II promoter, G1/S transition of the mitotic cell cycle, and negative regulation of DNA endoreduplication. A supplemental table of these genes and references regarding their possible functional roles in the cell cycle is provided (Supplementary Table S6).

With regards to the first gene ontology term (positive regulation of transcription from RNA polymerase II promoters), in other systems, these genes are involved in either directly promoting proliferation or facilitating proliferation by overcoming cellular defense mechanisms to cell cycle reentry (for a full set of references, see Supplementary Table S6). Overcoming cellular defense mechanisms activated in response to cell cycle reentry may be critical to achieving adult SC proliferation (Chen et al., 2003).

The genes categorized under the second gene ontology term (G1/S transition of the mitotic cell cycle) are related to the maintenance of quiescence in adult cochlear SCs (Malgrange et al., 2003; Chen and Segil, 1999; Laine et al., 2010; Oesterle et al., 2011). Transcript expression levels for these and other cell cycle-related genes may represent thresholds for cell cycle re-entry. Expression of these genes may be indicative of a poised state with modulation of these genes by subtle overexpression or inhibition around the baseline potentially leading to stimulation of cellular proliferation (Hu et al., 2016). This poised state has also been suggested by the discovery of bivalent chromatin structure in perinatal SCs (Stojanova et al., 2015; Hu et al., 2016) and may exist in adult SCs, albeit with additional barriers to regeneration in place.

Finally, with these additional barriers in mind, the genes categorized under negative regulation of DNA endoreduplication are related to facilitating but not necessarily initiating cell proliferation (see Supplementary Table S6 for a full set of references). Endoreduplication refers to re-replication of DNA within a single cell, likely representing a collection of compensatory mechanisms for maintaining the viability of cells that are unable to progress completely through the cell cycle (Lazzerini Denchi et al., 2006). Modulation of these genes may facilitate the creation of a genomic environment that is more conducive to cell proliferation, which under normal circumstances would be prevented/inhibited by several other factors.

Despite these analyses, it is possible that inducing adult SC proliferation will require the expression of cell cycle-related genes that are not normally transcribed by adult cochlear SCs. Furthermore, others have argued for the existence of epigenetic mechanisms preventing SCs from utilizing these machinery (Geng et al., 2016; Samarajeewa et al., 2019). Interactions between existing cell cycle gene expression and epigenetic machinery may maintain adult cochlear SC quiescence and remain incompletely elucidated. Nonetheless, the results presented here indicate the expression of a wide network of cell cycle genes in adult cochlear SCs, suggesting that a better understanding of the roles of these networks in maintaining adult SC quiescence could provide valuable insights regarding pathways to HC regeneration. Future studies will be required to test the relevance of modulating cell cycle gene expression or modulating interactions between cell cycle genes and epigenetic machinery to achieving HC regeneration in the adult cochlea. These data suggest that there are likely multiple mechanisms that may be involved in maintaining the latent potential of cochlear SCs to re-enter the cell cycle and to transdifferentiate into HCs. Understanding the potentially different avenues of overcoming this poised state in adult cochlear SCs may lead to more effective HC regeneration in the future.

## Conclusion

In summary, we have used several different approaches, including sc-RNAseq, ddPCR and sc-qPCR, to characterize and validate the transcriptional profiles of cochlear SCs from adult mice. Our results demonstrate significant changes in gene expression between perinatal and adult SCs. We provide some preliminary evidence that the patterns of expression observed in the adult mice may be comparable to human inner ears. Finally, analysis of gene expression in adult SCs indicates strong expression of pathways related to the regulation of the cell cycle, suggesting that targeting of these pathways could help to force cells out of quiescence. These results provide insights that could be relevant to the development of treatments to induce HC regeneration, which will, most likely, include the trans-differentiation of SCs into new HCs, leading to a deficit in SCs. If this is the case, controlled creation of new SCs, through cell cycle re-entry, may be a crucial step in the restoration of a functional cochlea.

## Data Availability Statement

The datasets generated for this study can be found in GEO (GEO Accession ID: GSE135703) and have been uploaded to gEAR and are available on this permalink (https://umgear.org/p?l=9f88d6fb).

## Ethics Statement

The studies involving human participants were reviewed and approved by University of California at Los Angeles Institutional Review Board (IRB protocol #10-001449). Appropriate informed consent for inclusion in the study was obtained from each temporal bone donor. Approval was obtained from the University of California at Los Angeles Institutional Review Board (IRB protocol #10-001449). The patients/participants provided their written informed consent to participate in this study. The animal study was reviewed and approved by The Animal Care and Use Committee of the National Institute of Neurological Diseases and Stroke and the National Institute on Deafness and Other Communication Disorders, National Institutes of Health.

## Author Contributions

MH and RO contributed to FACS, single-cell RNA-sequencing (scRNA-Seq), sc-qPCR and ddPCR. MH, RO, XL, AD, and IT contributed to immunohistochemistry and smFISH. IL, FL, RO, and MH contributed to human temporal bone histopathology. AI analyzed and selected pertinent archival histological material used for immunohistochemical staining and provided support for confocal prescreening of samples. DM and RM were responsible for scRNA-Seq. MH and IT were responsible for scRNA-Seq analysis. SG was responsible for the comparison of the scRNA-Seq dataset to existing datasets in the literature. MH, DM, RM, and MK contributed to writing and revising the manuscript. All authors read and approved the final manuscript.

## Conflict of Interest

The authors declare that the research was conducted in the absence of any commercial or financial relationships that could be construed as a potential conflict of interest.

## Footnotes

^1^https://satijalab.org/seurat/v3.1/pbmc3k_tutorial.html

^2^http://www.bio-rad.com/webroot/web/pdf/lsr/literature/10028376.pdf

^3^http://amp.pharm.mssm.edu/Enrichr/

^4^http://string-db.org

^5^https://satijalab.org/seurat/v3.1/cell_cycle_vignette.html
