# Corrigendum: Characterizing Adult Cochlear Supporting Cell Transcriptional Diversity Using Single-Cell RNA-Seq: Validation in the Adult Mouse and Translational Implications for the Adult Human Cochlea

**DOI:** 10.3389/fnmol.2021.699859

**Published:** 2021-06-03

**Authors:** Michael Hoa, Rafal Olszewski, Xiaoyi Li, Ian Taukulis, Shoujun Gu, Alvin DeTorres, Ivan A. Lopez, Fred H. Linthicum, Akira Ishiyama, Daniel Martin, Robert J. Morell, Matthew W. Kelley

**Affiliations:** ^1^Auditory Restoration and Development Program, National Institute on Deafness and Other Communication Disorders, NIH, Bethesda, MD, United States; ^2^National Temporal Bone Laboratory at UCLA, UCLA School of Medicine, Los Angeles, CA, United States; ^3^Cellular and Molecular Biology of the Inner Ear Laboratory, UCLA School of Medicine, Los Angeles, CA, United States; ^4^Biomedical Research Informatics Office, National Institute of Dental and Craniofacial Research, NIH, Bethesda, MD, United States; ^5^Genomics and Computational Biology Core, National Institute on Deafness and Other Communication Disorders, NIH, Bethesda, MD, United States; ^6^Laboratory of Cochlear Development, National Institute on Deafness and Other Communication Disorders, NIH, Bethesda, MD, United States

**Keywords:** inner ear, supporting cell subtypes, smFISH, adult (MeSH), cell cycle, FACS, cochlea

In the original article, there was a mistake in the Supplementary Material file “Data_Sheet_2_Characterizing Adult Cochlear Supporting Cell Transcriptional Diversity Using Single-Cell RNA-Seq_Validation in the Adult Mouse and Tran,” which contains Supplemental Figures S14 to S24, where the *p*-value appeared to be greater than 1 in some cases. This was due to a multiplier being cut off from the column display. In cases, where the *p*-value is 0.0 this indicates that the *p*-value <0.0001.

The authors apologize for this error and this does not change the scientific conclusions of the article in any way. The original supplementary material has been updated.

