# Supplementary material for: Characterizing Adult Cochlear Supporting Cell Transcriptional Diversity Using Single-Cell RNA-Seq: Validation in the Adult Mouse and Translational Implications for the Adult Human Cochlea
Source: Front Mol Neurosci. 2020 Feb 5;13:13. doi: 10.3389/fnmol.2020.00013 (PMC7012811; doi:10.3389/fnmol.2020.00013)
Supplement: Supplementary file 20 [file Data_Sheet_1.PDF]

Supplemental Datasheet S1. Preliminary evidence for adult mouse supporting cell-specific gene expression in human inner ears.

Results

Preliminary evidence for adult mouse supporting cell-specific gene expression in human temporal bones. Prior to attempting immunolocalization in human specimens, we validated antibodies for S100A6 and LCP1 in adult mouse organ of Corti, as smFISH has not been previously demonstrated to work in archival temporal bone specimens. S100A6 protein is expressed in predominantly medial SCs (border and inner phalangeal cells) with some expression noted in the Hensen's cells in the adult mouse as seen on mid-modiolar cross-sections of the adult organ of Corti (Supplemental Figure S8A-A'). Grayscale single channel images of S100A6 and LCP1 protein expression are shown in Supplemental Figures S8A' and S8B', respectively. S100A6 protein expression in Hensen's cells appears to be intermediate to expression in inner border/inner phalangeal cells and Deiters cells, respectively. LCP1 protein was localized predominantly to the pillar cells (Supplemental Figure S8B-B'). This differs from the results of smFISH in Figure 2 where the distribution of *Lcp1* RNA transcripts is observed in all cochlear supporting cells (Figure 2E). This observed incongruence between *Lcp1* RNA and LCP1 protein could be due to undefined post-transcriptional processing mechanisms, differences in the half-life of the LCP1 protein in vivo, or imperfect binding of the antibody to the LCP1 protein (Greenbaum et al. 2003).

An antibody against acetylated tubulin was utilized to label adult human cochlear supporting cells and confirm cell type-specific expression in human samples. A brightfield image of a human organ of Corti is shown in Supplemental Figure S8C with an arrow pointing to the region of the inner hair cell and a bracket outlining the outer hair cell region. S100A6 and LCP1 expression are demonstrated in adult human cochlear supporting cells and co-localize with acetylated tubulin, a known adult supporting cell marker (Supplemental Figure S8D-D", S8F-F"). Unlike S100A6 protein in mouse, S100A6 protein in humans appears to be expressed by all cochlear supporting cells. Between mouse and human, LCP1 protein expression appears to correlate with expression noted in the pillar cells. Unlike acetylated tubulin, which is expressed in the human stria vascularis similar to observations in the mouse stria vascularis (Liu et al. 2018), LCP1 is not expressed in the stria vascularis (Supplemental Figure S9). Representative negative control images showing secondary antibody only and unlabeled slides to account for residual autofluorescence are provided (Supplemental Figure S10). These data demonstrate that S100A6 and LCP1 protein are expressed by adult human cochlear SCs. Overall, our data suggest that other supporting cell markers identified in the single cell transcriptome dataset of adult mouse cochlear SCs could be reliable markers for adult human SCs.

Discussion

Preliminary evidence for adult mouse cochlear supporting cell-specific gene expression in human inner ears. An important consideration in any biomedically-related study using an animal model is the applicability to humans. For this study, we examined the expression of two candidate adult mouse SC genes in human cochlear SCs. S100A6 and LCP1 demonstrated slightly different

patterns of expression in humans versus mice. Specifically, S100A6 protein appears to be expressed in most human supporting cells (Supplemental Figure S8D-D"). This contrasts with the mouse cochlea where S100A6 protein immunostaining is more prominent in medial SCs (Supplemental Figure S8A-A'). In addition to transcriptional differences between mouse and humans, S100A6 is known to be secreted and taken up by other cells, which may explain this apparent disparity in protein expression between mouse and human SCs (Jurewicz, Wyroba, and Filipek 2018). In contrast to the observed broad expression *Lcp1* RNA across all supporting cells in the mouse (Figure 2F), LCP1 protein expression in the mouse and human organ of Corti appears to be confined to the pillar cells (Supplemental Figure S8B-B', S8E-E'). Potential reasons for this disparity include insufficiently defined post-transcriptional mechanisms involved in converting mRNA into protein, differing in vivo half-lives of protein, and the existence of a significant amount of error and noise in both protein and mRNA experiments (Greenbaum et al. 2003). Greenbaum and colleagues also suggest that changes in the rate of protein and mRNA synthesis as well as protein turnover may contribute to this disparity. These data point to the utility of human temporal bone repositories as a resource to validate and confirm candidates for hair cell regeneration attempts in humans.

References

Greenbaum, Dov, Christopher Colangelo, Kenneth Williams, and Mark Gerstein. 2003. "Comparing Protein Abundance and MRNA Expression Levels on a Genomic Scale." *Genome Biology*. https://doi.org/10.1186/gb-2003-4-9-117.

Jurewicz, Ewelina, Elżbieta Wyroba, and Anna Filipek. 2018. "Tubulin-Dependent Secretion of S100A6 and Cellular Signaling Pathways Activated by S100A6-Integrin B1 Interaction." *Cellular Signalling* 42 (January): 21–29. https://doi.org/10.1016/j.cellsig.2017.10.004.

Liu, Wen Jing, Chuan Xi Wang, Hao Yu, Shao Feng Liu, and Jun Yang. 2018. "Expression of Acetylated Tubulin in the Postnatal Developing Mouse Cochlea." *European Journal of Histochemistry*. https://doi.org/10.4081/ejh.2018.2942.
