# Supplementary material for: Characterizing Adult Cochlear Supporting Cell Transcriptional Diversity Using Single-Cell RNA-Seq: Validation in the Adult Mouse and Translational Implications for the Adult Human Cochlea
Source: Front Mol Neurosci. 2020 Feb 5;13:13. doi: 10.3389/fnmol.2020.00013 (PMC7012811; doi:10.3389/fnmol.2020.00013)
Supplement: Supplementary file 21 [file Data_Sheet_2.PDF]

Supplemental Figure S14. Comparison to genes preferentially expressed in Deiters cells compared to IHC (Liu et al. 2018, Li et al, 2018)

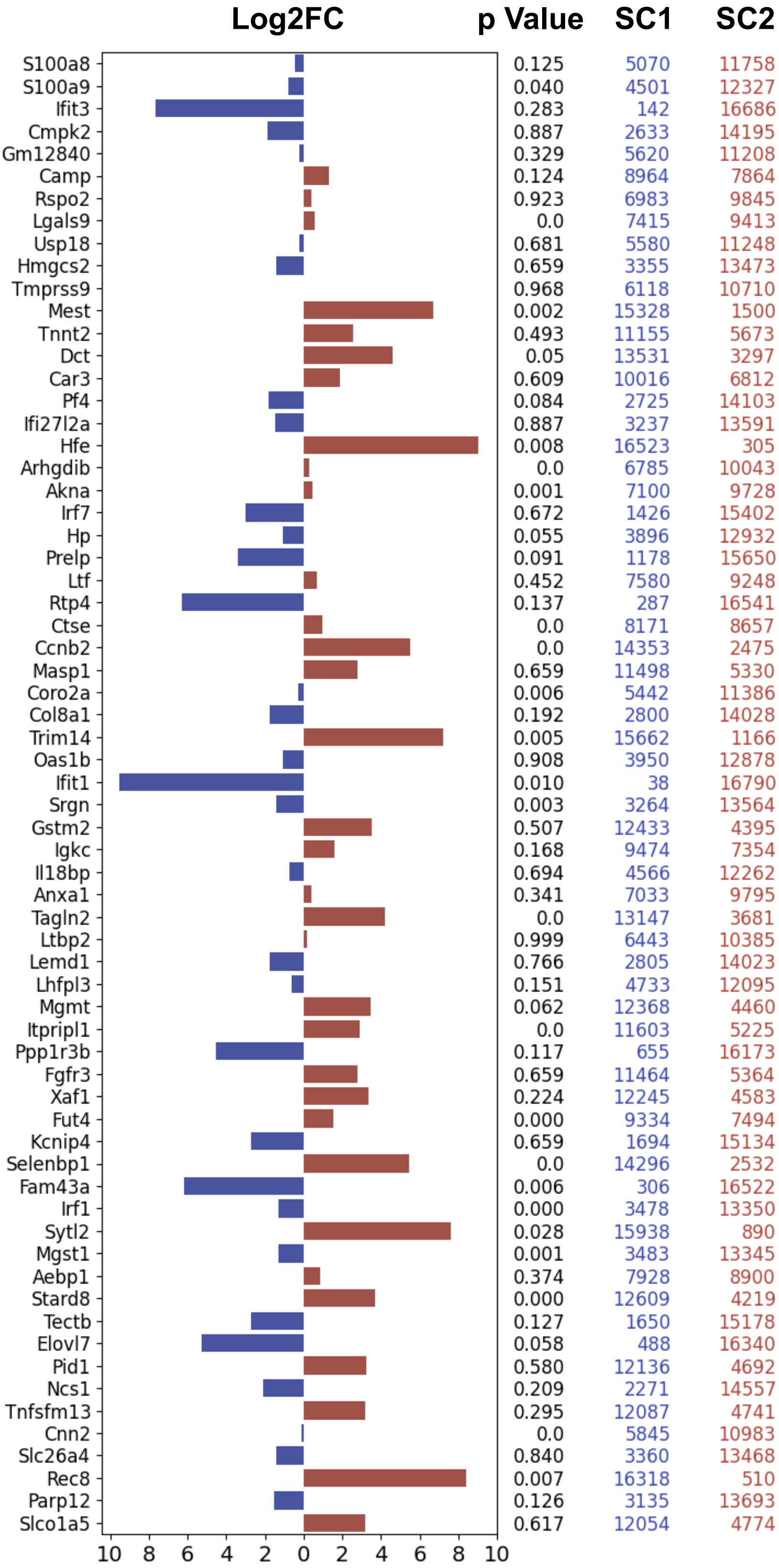

Gene	p Value	SC1	SC2
S100a8	0.125	5070	11758
S100a9	0.040	4501	12327
Ifit3	0.283	142	16686
Cmpk2	0.887	2633	14195
Gm12840	0.329	5620	11208
Camp	0.124	8964	7864
Rspo2	0.923	6983	9845
Lgals9	0.0	7415	9413
Usp18	0.681	5580	11248
Hmgcs2	0.659	3355	13473
Tmprss9	0.968	6118	10710
Mest	0.002	15328	1500
Tnnt2	0.493	11155	5673
Dct	0.05	13531	3297
Car3	0.609	10016	6812
Pf4	0.084	2725	14103
Ifi27l2a	0.887	3237	13591
Hfe	0.008	16523	305
Arhgdib	0.0	6785	10043
Akna	0.001	7100	9728
Irf7	0.672	1426	15402
Hp	0.055	3896	12932
Prelp	0.091	1178	15650
Ltf	0.452	7580	9248
Rtp4	0.137	287	16541
Ctse	0.0	8171	8657
Ccnb2	0.0	14353	2475
Masp1	0.659	11498	5330
Coro2a	0.006	5442	11386
Col8a1	0.192	2800	14028
Trim14	0.005	15662	1166
Oas1b	0.908	3950	12878
Ifit1	0.010	38	16790
Srgn	0.003	3264	13564
Gstm2	0.507	12433	4395
Igkc	0.168	9474	7354
Il18bp	0.694	4566	12262
Anxa1	0.341	7033	9795
Tagln2	0.0	13147	3681
Ltbp2	0.999	6443	10385
Lemd1	0.766	2805	14023
Lhfpl3	0.151	4733	12095
Mgmt	0.062	12368	4460
Itpripl1	0.0	11603	5225
Ppp1r3b	0.117	655	16173
Fgfr3	0.659	11464	5364
Xaf1	0.224	12245	4583
Fut4	0.000	9334	7494
Kcnip4	0.659	1694	15134
Selenbp1	0.0	14296	2532
Fam43a	0.006	306	16522
Irf1	0.000	3478	13350
Sytl2	0.028	15938	890
Mgst1	0.001	3483	13345
Aebp1	0.374	7928	8900
Stard8	0.000	12609	4219
Tectb	0.127	1650	15178
Elovl7	0.058	488	16340
Pid1	0.580	12136	4692
Ncs1	0.209	2271	14557
Tnfsfm13	0.295	12087	4741
Cnn2	0.0	5845	10983
Slc26a4	0.840	3360	13468
Rec8	0.007	16318	510
Parp12	0.126	3135	13693
Slco1a5	0.617	12054	4774

*Numbers under SC1 and SC2 columns represent the gene rank within SC1 and SC2 data subsets, respectively. This convention applies for Supplemental Figures S11-S21.

Supplemental Figure S15. Comparison to genes preferentially expressed in Deiters cells compared to OHC (Liu et al. 2018, Li et al, 2018)

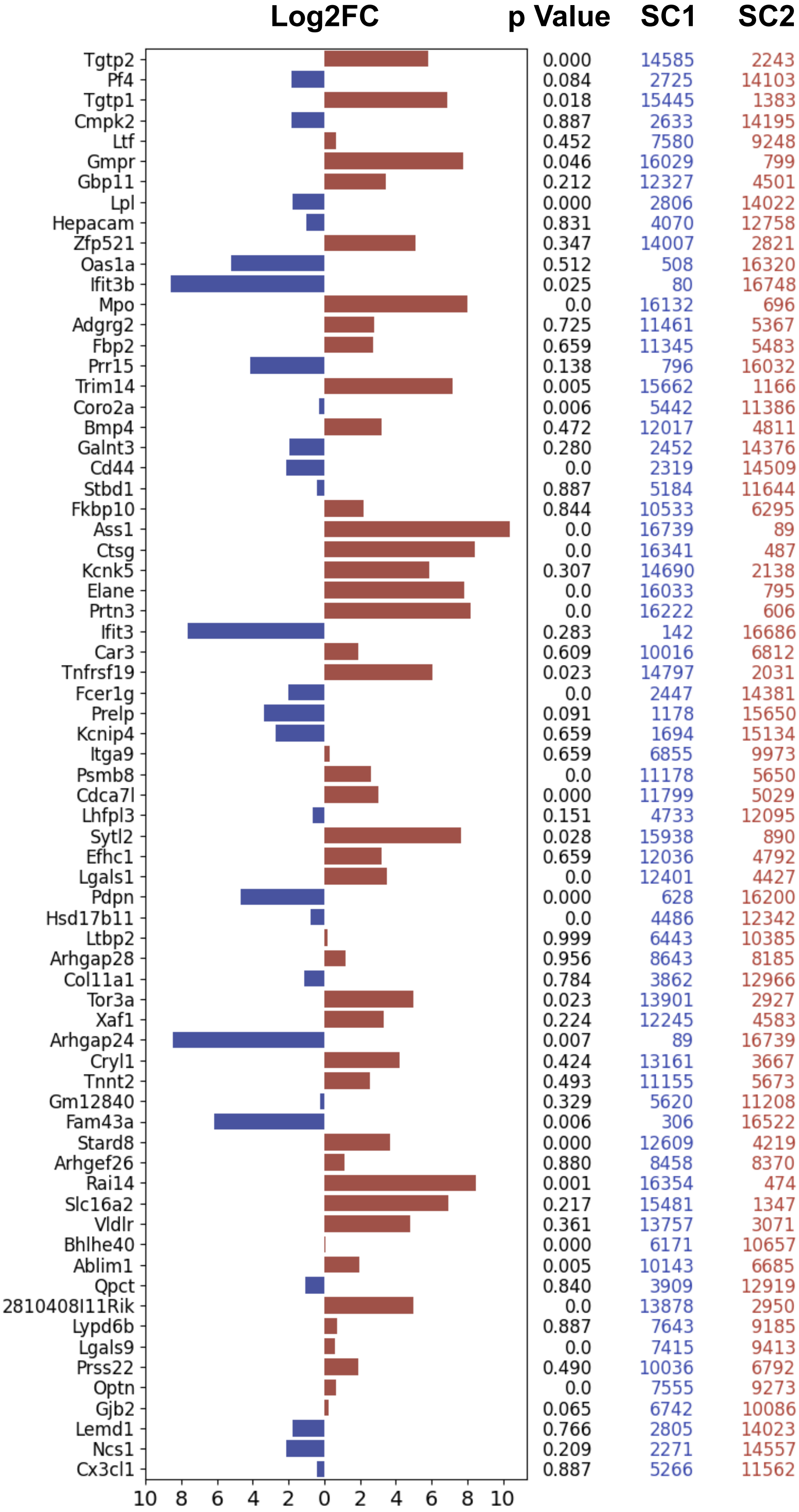

Gene	p Value	SC1	SC2
Tgtp2	0.000	14585	2243
Pf4	0.084	2725	14103
Tgtp1	0.018	15445	1383
Cmpk2	0.887	2633	14195
Ltf	0.452	7580	9248
Gmpr	0.046	16029	799
Gbp11	0.212	12327	4501
Lpl	0.000	2806	14022
Hepacam	0.831	4070	12758
Zfp521	0.347	14007	2821
Oas1a	0.512	508	16320
Ifit3b	0.025	80	16748
Mpo	0.0	16132	696
Adgrg2	0.725	11461	5367
Fbp2	0.659	11345	5483
Prr15	0.138	796	16032
Trim14	0.005	15662	1166
Coro2a	0.006	5442	11386
Bmp4	0.472	12017	4811
Galnt3	0.280	2452	14376
Cd44	0.0	2319	14509
Stbd1	0.887	5184	11644
Fkbp10	0.844	10533	6295
Ass1	0.0	16739	89
Ctsg	0.0	16341	487
Kcnk5	0.307	14690	2138
Elane	0.0	16033	795
Prtn3	0.0	16222	606
Ifit3	0.283	142	16686
Car3	0.609	10016	6812
Tnfrsf19	0.023	14797	2031
Fcer1g	0.0	2447	14381
Prelp	0.091	1178	15650
Kcnip4	0.659	1694	15134
Itga9	0.659	6855	9973
Psmb8	0.0	11178	5650
Cdca7l	0.000	11799	5029
Lhfpl3	0.151	4733	12095
Sytl2	0.028	15938	890
Efhc1	0.659	12036	4792
Lgals1	0.0	12401	4427
Pdpn	0.000	628	16200
Hsd17b11	0.0	4486	12342
Ltbp2	0.999	6443	10385
Arhgap28	0.956	8643	8185
Col11a1	0.784	3862	12966
Tor3a	0.023	13901	2927
Xaf1	0.224	12245	4583
Arhgap24	0.007	89	16739
Cryl1	0.424	13161	3667
Tnnt2	0.493	11155	5673
Gm12840	0.329	5620	11208
Fam43a	0.006	306	16522
Stard8	0.000	12609	4219
Arhgef26	0.880	8458	8370
Rai14	0.001	16354	474
Slc16a2	0.217	15481	1347
Vldlr	0.361	13757	3071
Bhlhe40	0.000	6171	10657
Ablim1	0.005	10143	6685
Qpct	0.840	3909	12919
2810408I11Rik	0.0	13878	2950
Lypd6b	0.887	7643	9185
Lgals9	0.0	7415	9413
Prss22	0.490	10036	6792
Optn	0.0	7555	9273
Gjb2	0.065	6742	10086
Lemd1	0.766	2805	14023
Ncs1	0.209	2271	14557
Cx3cl1	0.887	5266	11562

Supplemental Figure S16. Comparison to genes preferentially expressed in Deiters cells compared to pillar cells (Liu et al. 2018)

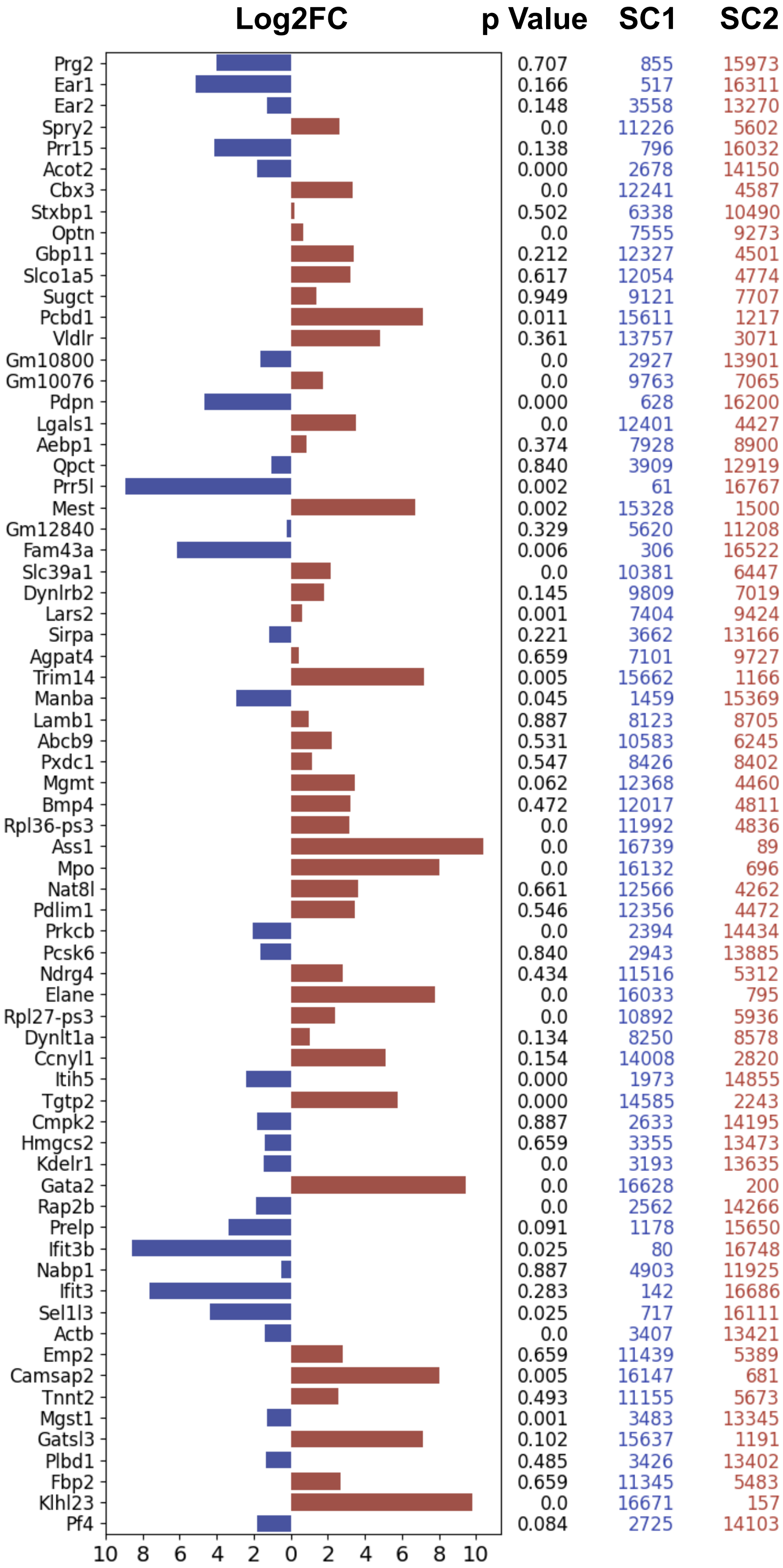

Gene	p Value	SC1	SC2
Prg2	0.707	855	15973
Ear1	0.166	517	16311
Ear2	0.148	3558	13270
Spry2	0.0	11226	5602
Prr15	0.138	796	16032
Acot2	0.000	2678	14150
Cbx3	0.0	12241	4587
Stxbp1	0.502	6338	10490
Optn	0.0	7555	9273
Gbp11	0.212	12327	4501
Slco1a5	0.617	12054	4774
Sugct	0.949	9121	7707
Pcbd1	0.011	15611	1217
Vldlr	0.361	13757	3071
Gm10800	0.0	2927	13901
Gm10076	0.0	9763	7065
Pdpn	0.000	628	16200
Lgals1	0.0	12401	4427
Aebp1	0.374	7928	8900
Qpct	0.840	3909	12919
Prr5l	0.002	61	16767
Mest	0.002	15328	1500
Gm12840	0.329	5620	11208
Fam43a	0.006	306	16522
Slc39a1	0.0	10381	6447
Dynlrb2	0.145	9809	7019
Lars2	0.001	7404	9424
Sirpa	0.221	3662	13166
Agpat4	0.659	7101	9727
Trim14	0.005	15662	1166
Manba	0.045	1459	15369
Lamb1	0.887	8123	8705
Abcb9	0.531	10583	6245
Pxdc1	0.547	8426	8402
Mgmt	0.062	12368	4460
Bmp4	0.472	12017	4811
Rpl36-ps3	0.0	11992	4836
Ass1	0.0	16739	89
Mpo	0.0	16132	696
Nat8l	0.661	12566	4262
Pdlim1	0.546	12356	4472
Prkcb	0.0	2394	14434
Pcsk6	0.840	2943	13885
Ndrg4	0.434	11516	5312
Elane	0.0	16033	795
Rpl27-ps3	0.0	10892	5936
Dynlt1a	0.134	8250	8578
Ccnyl1	0.154	14008	2820
Itih5	0.000	1973	14855
Tgtp2	0.000	14585	2243
Cmpk2	0.887	2633	14195
Hmgcs2	0.659	3355	13473
Kdelr1	0.0	3193	13635
Gata2	0.0	16628	200
Rap2b	0.0	2562	14266
Prelp	0.091	1178	15650
Ifit3b	0.025	80	16748
Nabp1	0.887	4903	11925
Ifit3	0.283	142	16686
Sel1l3	0.025	717	16111
Actb	0.0	3407	13421
Emp2	0.659	11439	5389
Camsap2	0.005	16147	681
Tnnt2	0.493	11155	5673
Mgst1	0.001	3483	13345
Gatsl3	0.102	15637	1191
Plbd1	0.485	3426	13402
Fbp2	0.659	11345	5483
Klhl23	0.0	16671	157
Pf4	0.084	2725	14103

Supplemental Figure S17. Comparison to genes preferentially expressed in Pillar cells compared to Deiter cells (Liu et al. 2018)

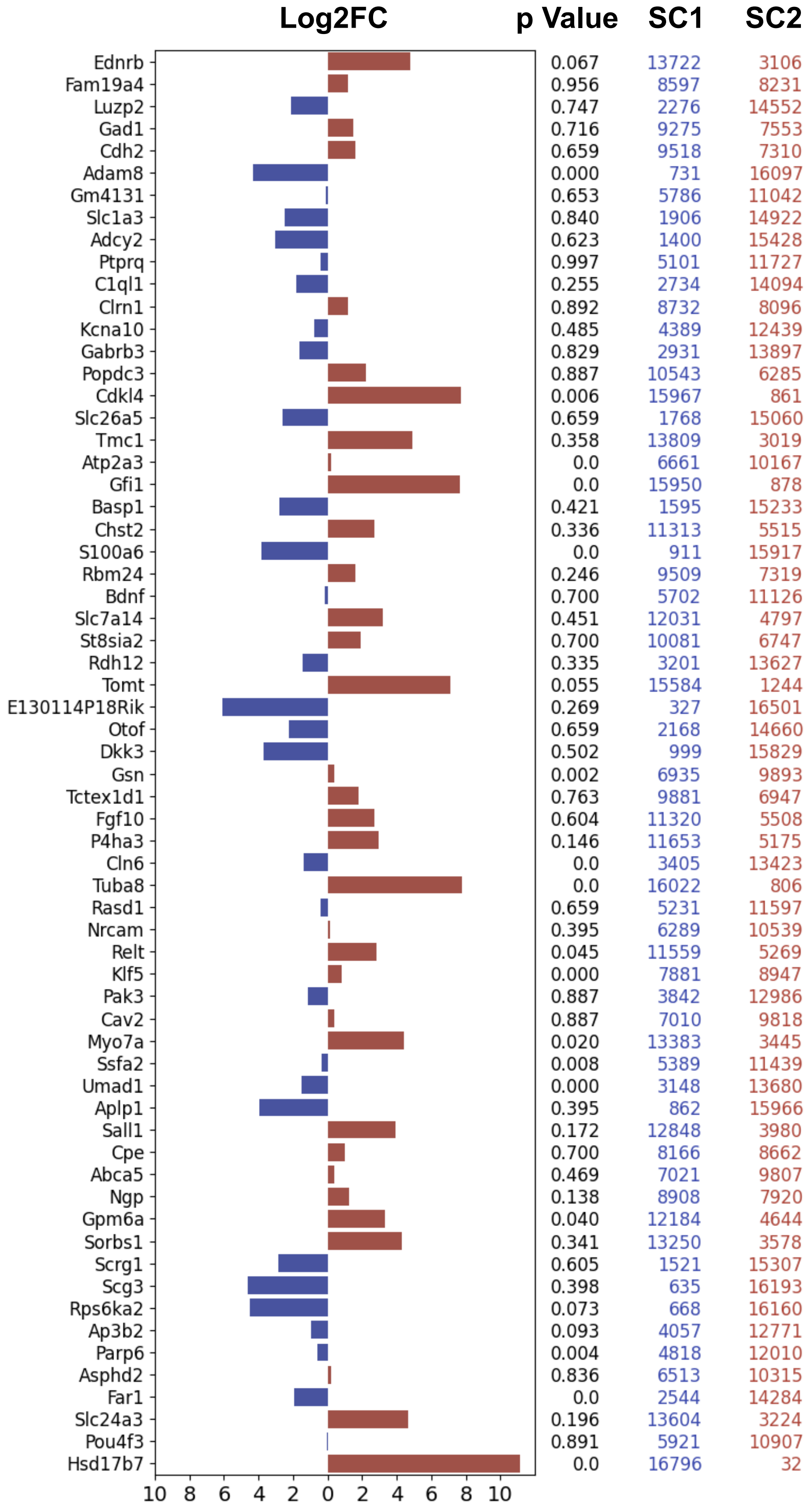

Gene	p Value	SC1	SC2
Ednrb	0.067	13722	3106
Fam19a4	0.956	8597	8231
Luzp2	0.747	2276	14552
Gad1	0.716	9275	7553
Cdh2	0.659	9518	7310
Adam8	0.000	731	16097
Gm4131	0.653	5786	11042
Slc1a3	0.840	1906	14922
Adcy2	0.623	1400	15428
Ptprq	0.997	5101	11727
C1ql1	0.255	2734	14094
Clrn1	0.892	8732	8096
Kcna10	0.485	4389	12439
Gabrb3	0.829	2931	13897
Popdc3	0.887	10543	6285
Cdkl4	0.006	15967	861
Slc26a5	0.659	1768	15060
Tmc1	0.358	13809	3019
Atp2a3	0.0	6661	10167
Gfi1	0.0	15950	878
Basp1	0.421	1595	15233
Chst2	0.336	11313	5515
S100a6	0.0	911	15917
Rbm24	0.246	9509	7319
Bdnf	0.700	5702	11126
Slc7a14	0.451	12031	4797
St8sia2	0.700	10081	6747
Rdh12	0.335	3201	13627
Tomt	0.055	15584	1244
E130114P18Rik	0.269	327	16501
Otof	0.659	2168	14660
Dkk3	0.502	999	15829
Gsn	0.002	6935	9893
Tctex1d1	0.763	9881	6947
Fgf10	0.604	11320	5508
P4ha3	0.146	11653	5175
Cln6	0.0	3405	13423
Tuba8	0.0	16022	806
Rasd1	0.659	5231	11597
Nrcam	0.395	6289	10539
Relt	0.045	11559	5269
Klf5	0.000	7881	8947
Pak3	0.887	3842	12986
Cav2	0.887	7010	9818
Myo7a	0.020	13383	3445
Ssfa2	0.008	5389	11439
Umad1	0.000	3148	13680
Aplp1	0.395	862	15966
Sall1	0.172	12848	3980
Cpe	0.700	8166	8662
Abca5	0.469	7021	9807
Ngp	0.138	8908	7920
Gpm6a	0.040	12184	4644
Sorbs1	0.341	13250	3578
Scrg1	0.605	1521	15307
Scg3	0.398	635	16193
Rps6ka2	0.073	668	16160
Ap3b2	0.093	4057	12771
Parp6	0.004	4818	12010
Asphd2	0.836	6513	10315
Far1	0.0	2544	14284
Slc24a3	0.196	13604	3224
Pou4f3	0.891	5921	10907
Hsd17b7	0.0	16796	32

Supplemental Figure S18. Comparison to genes preferentially expressed in Pillar cells compared to IHC (Liu et al. 2018; Li et al, 2018)

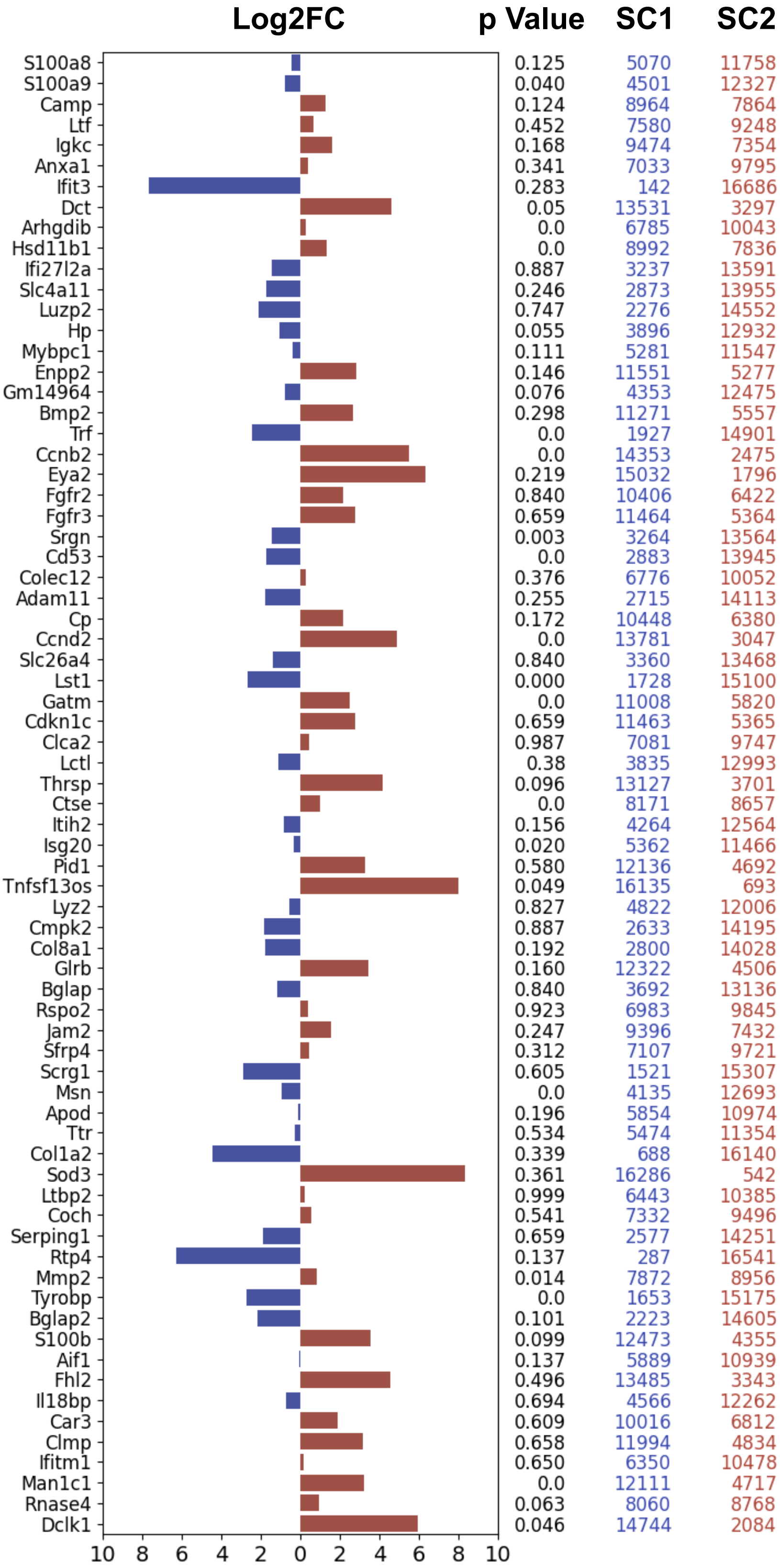

Gene	p Value	SC1	SC2
S100a8	0.125	5070	11758
S100a9	0.040	4501	12327
Camp	0.124	8964	7864
Ltf	0.452	7580	9248
Igkc	0.168	9474	7354
Anxa1	0.341	7033	9795
Ifit3	0.283	142	16686
Dct	0.05	13531	3297
Arhgdib	0.0	6785	10043
Hsd11b1	0.0	8992	7836
Ifi27l2a	0.887	3237	13591
Slc4a11	0.246	2873	13955
Luzp2	0.747	2276	14552
Hp	0.055	3896	12932
Mybpc1	0.111	5281	11547
Enpp2	0.146	11551	5277
Gm14964	0.076	4353	12475
Bmp2	0.298	11271	5557
Trf	0.0	1927	14901
Ccnb2	0.0	14353	2475
Eya2	0.219	15032	1796
Fgfr2	0.840	10406	6422
Fgfr3	0.659	11464	5364
Srgn	0.003	3264	13564
Cd53	0.0	2883	13945
Colec12	0.376	6776	10052
Adam11	0.255	2715	14113
Cp	0.172	10448	6380
Ccnd2	0.0	13781	3047
Slc26a4	0.840	3360	13468
Lst1	0.000	1728	15100
Gatm	0.0	11008	5820
Cdkn1c	0.659	11463	5365
Clca2	0.987	7081	9747
Lctl	0.38	3835	12993
Thrsp	0.096	13127	3701
Ctse	0.0	8171	8657
Itih2	0.156	4264	12564
Isg20	0.020	5362	11466
Pid1	0.580	12136	4692
Tnfsf13os	0.049	16135	693
Lyz2	0.827	4822	12006
Cmpk2	0.887	2633	14195
Col8a1	0.192	2800	14028
Glrb	0.160	12322	4506
Bglap	0.840	3692	13136
Rspo2	0.923	6983	9845
Jam2	0.247	9396	7432
Sfrp4	0.312	7107	9721
Scrg1	0.605	1521	15307
Msn	0.0	4135	12693
Apod	0.196	5854	10974
Ttr	0.534	5474	11354
Col1a2	0.339	688	16140
Sod3	0.361	16286	542
Ltbp2	0.999	6443	10385
Coch	0.541	7332	9496
Serping1	0.659	2577	14251
Rtp4	0.137	287	16541
Mmp2	0.014	7872	8956
Tyrobp	0.0	1653	15175
Bglap2	0.101	2223	14605
S100b	0.099	12473	4355
Aif1	0.137	5889	10939
Fhl2	0.496	13485	3343
Il18bp	0.694	4566	12262
Car3	0.609	10016	6812
Clmp	0.658	11994	4834
Ifitm1	0.650	6350	10478
Man1c1	0.0	12111	4717
Rnase4	0.063	8060	8768
Dclk1	0.046	14744	2084

Supplemental Figure S19. Comparison to genes preferentially expressed in Pillar cells compared to OHC (Liu et al. 2018; Li et al. 2018)

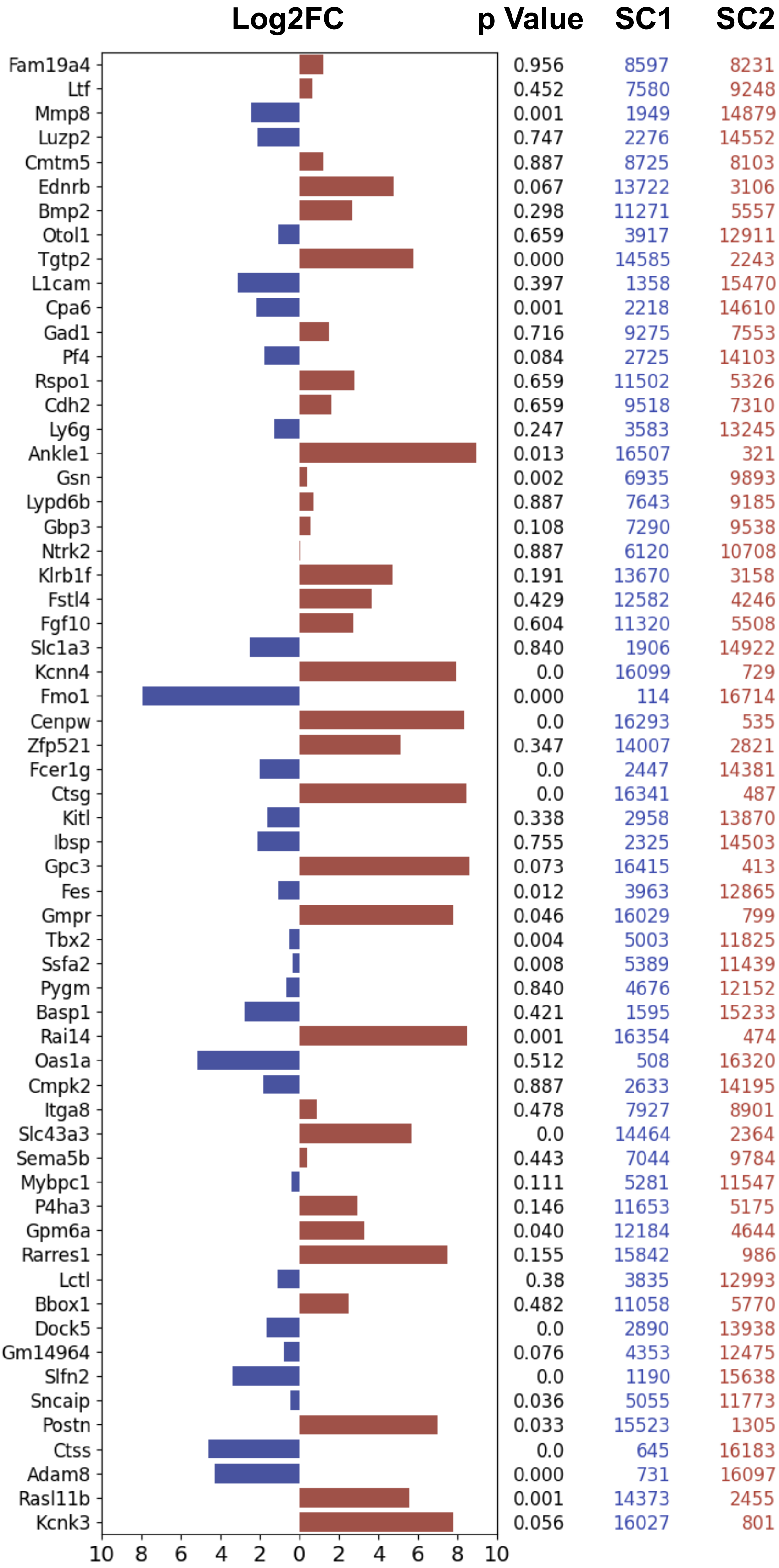

Gene	p Value	SC1	SC2
Fam19a4	0.956	8597	8231
Ltf	0.452	7580	9248
Mmp8	0.001	1949	14879
Luzp2	0.747	2276	14552
Cmtm5	0.887	8725	8103
Ednrb	0.067	13722	3106
Bmp2	0.298	11271	5557
Otol1	0.659	3917	12911
Tgtp2	0.000	14585	2243
L1cam	0.397	1358	15470
Cpa6	0.001	2218	14610
Gad1	0.716	9275	7553
Pf4	0.084	2725	14103
Rspo1	0.659	11502	5326
Cdh2	0.659	9518	7310
Ly6g	0.247	3583	13245
Ankle1	0.013	16507	321
Gsn	0.002	6935	9893
Lypd6b	0.887	7643	9185
Gbp3	0.108	7290	9538
Ntrk2	0.887	6120	10708
Klrb1f	0.191	13670	3158
Fstl4	0.429	12582	4246
Fgf10	0.604	11320	5508
Slc1a3	0.840	1906	14922
Kcnn4	0.0	16099	729
Fmo1	0.000	114	16714
Cenpw	0.0	16293	535
Zfp521	0.347	14007	2821
Fcer1g	0.0	2447	14381
Ctsg	0.0	16341	487
Kitl	0.338	2958	13870
Ibsp	0.755	2325	14503
Gpc3	0.073	16415	413
Fes	0.012	3963	12865
Gmpr	0.046	16029	799
Tbx2	0.004	5003	11825
Ssfa2	0.008	5389	11439
Pygm	0.840	4676	12152
Basp1	0.421	1595	15233
Rai14	0.001	16354	474
Oas1a	0.512	508	16320
Cmpk2	0.887	2633	14195
Itga8	0.478	7927	8901
Slc43a3	0.0	14464	2364
Sema5b	0.443	7044	9784
Mybpc1	0.111	5281	11547
P4ha3	0.146	11653	5175
Gpm6a	0.040	12184	4644
Rarres1	0.155	15842	986
Lctl	0.38	3835	12993
Bbox1	0.482	11058	5770
Dock5	0.0	2890	13938
Gm14964	0.076	4353	12475
Slfn2	0.0	1190	15638
Sncaip	0.036	5055	11773
Postn	0.033	15523	1305
Ctss	0.0	645	16183
Adam8	0.000	731	16097
Rasl11b	0.001	14373	2455
Kcnk3	0.056	16027	801

Supplemental Figure S20. Comparison to IHC (Liu et al. 2014; Li et al. 2018)

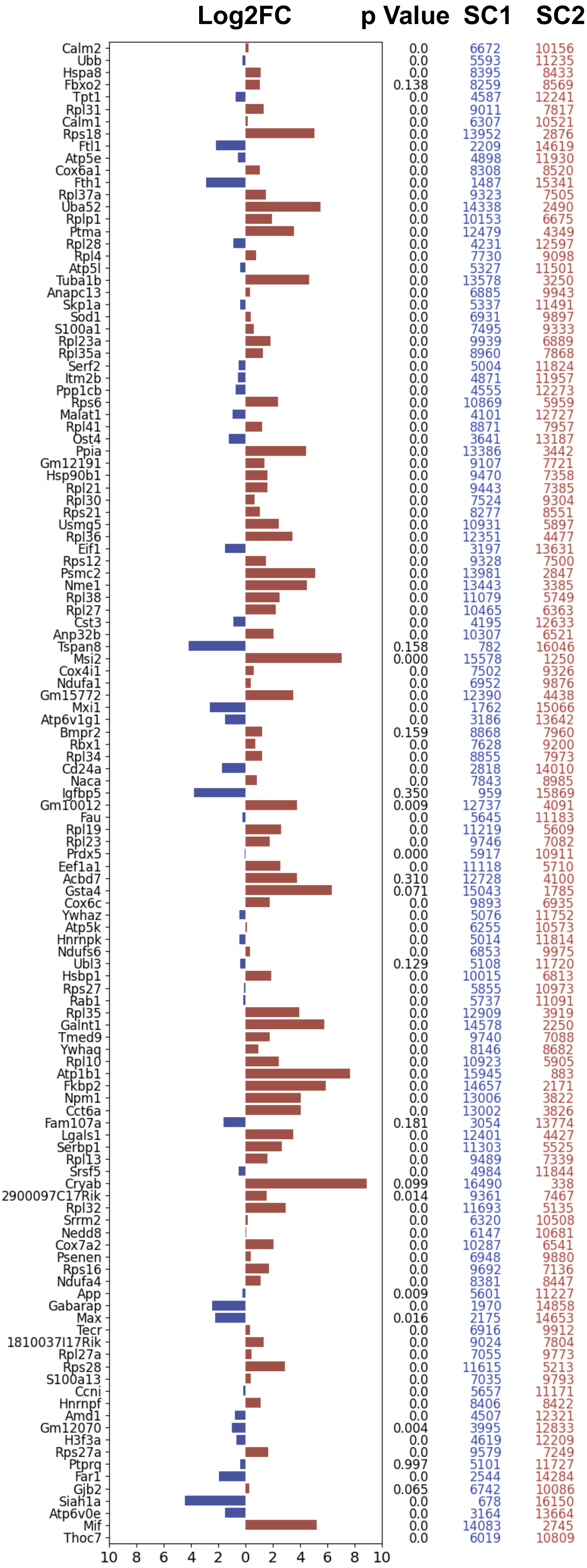

Gene	p Value	SC1	SC2
Calm2	0.0	6672	10156
Ubb	0.0	5593	11235
Hspa8	0.0	8395	8433
Fbxo2	0.138	8259	8569
Tpt1	0.0	4587	12241
Rpl31	0.0	9011	7817
Calm1	0.0	6307	10521
Rps18	0.0	13952	2876
Ftl1	0.0	2209	14619
Atp5e	0.0	4898	11930
Cox6a1	0.0	8308	8520
Fth1	0.0	1487	15341
Rpl37a	0.0	9323	7505
Uba52	0.0	14338	2490
Rplp1	0.0	10153	6675
Ptma	0.0	12479	4349
Rpl28	0.0	4231	12597
Rpl4	0.0	7730	9098
Atp5l	0.0	5327	11501
Tuba1b	0.0	13578	3250
Anapc13	0.0	6885	9943
Skp1a	0.0	5337	11491
Sod1	0.0	6931	9897
S100a1	0.0	7495	9333
Rpl23a	0.0	9939	6889
Rpl35a	0.0	8960	7868
Serf2	0.0	5004	11824
Itm2b	0.0	4871	11957
Ppp1cb	0.0	4555	12273
Rps6	0.0	10869	5959
Malat1	0.0	4101	12727
Rpl41	0.0	8871	7957
Ost4	0.0	3641	13187
Ppia	0.0	13386	3442
Gm12191	0.0	9107	7721
Hsp90b1	0.0	9470	7358
Rpl21	0.0	9443	7385
Rpl30	0.0	7524	9304
Rps21	0.0	8277	8551
Usmg5	0.0	10931	5897
Rpl36	0.0	12351	4477
Eif1	0.0	3197	13631
Rps12	0.0	9328	7500
Psmc2	0.0	13981	2847
Nme1	0.0	13443	3385
Rpl38	0.0	11079	5749
Rpl27	0.0	10465	6363
Cst3	0.0	4195	12633
Anp32b	0.0	10307	6521
Tspan8	0.158	782	16046
Msi2	0.000	15578	1250
Cox4i1	0.0	7502	9326
Ndufa1	0.0	6952	9876
Gm15772	0.0	12390	4438
Mxi1	0.0	1762	15066
Atp6v1g1	0.0	3186	13642
Bmpr2	0.159	8868	7960
Rbx1	0.0	7628	9200
Rpl34	0.0	8855	7973
Cd24a	0.0	2818	14010
Naca	0.0	7843	8985
Igfbp5	0.350	959	15869
Gm10012	0.009	12737	4091
Fau	0.0	5645	11183
Rpl19	0.0	11219	5609
Rpl23	0.0	9746	7082
Prdx5	0.000	5917	10911
Eef1a1	0.0	11118	5710
Acbd7	0.310	12728	4100
Gsta4	0.071	15043	1785
Cox6c	0.0	9893	6935
Ywhaz	0.0	5076	11752
Atp5k	0.0	6255	10573
Hnrnpk	0.0	5014	11814
Ndufs6	0.0	6853	9975
Ubl3	0.129	5108	11720
Hsbp1	0.0	10015	6813
Rps27	0.0	5855	10973
Rab1	0.0	5737	11091
Rpl35	0.0	12909	3919
Galnt1	0.0	14578	2250
Tmed9	0.0	9740	7088
Ywhaq	0.0	8146	8682
Rpl10	0.0	10923	5905
Atp1b1	0.0	15945	883
Fkbp2	0.0	14657	2171
Npm1	0.0	13006	3822
Cct6a	0.0	13002	3826
Fam107a	0.181	3054	13774
Lgals1	0.0	12401	4427
Serbp1	0.0	11303	5525
Rpl13	0.0	9489	7339
Srsf5	0.0	4984	11844
Cryab	0.099	16490	338
2900097C17Rik	0.014	9361	7467
Rpl32	0.0	11693	5135
Srrm2	0.0	6320	10508
Nedd8	0.0	6147	10681
Cox7a2	0.0	10287	6541
Psenen	0.0	6948	9880
Rps16	0.0	9692	7136
Ndufa4	0.0	8381	8447
App	0.009	5601	11227
Gabarap	0.0	1970	14858
Max	0.016	2175	14653
Tecr	0.0	6916	9912
1810037I17Rik	0.0	9024	7804
Rpl27a	0.0	7055	9773
Rps28	0.0	11615	5213
S100a13	0.0	7035	9793
Ccni	0.0	5657	11171
Hnrnpf	0.0	8406	8422
Amd1	0.0	4507	12321
Gm12070	0.004	3995	12833
H3f3a	0.0	4619	12209
Rps27a	0.0	9579	7249
Ptprq	0.997	5101	11727
Far1	0.0	2544	14284
Gjb2	0.065	6742	10086
Siah1a	0.0	678	16150
Atp6v0e	0.0	3164	13664
Mif	0.0	14083	2745
Thoc7	0.0	6019	10809

Supplemental Figure S21. Comparison to OHC (Liu et al. 2014; Li et al. 2018)

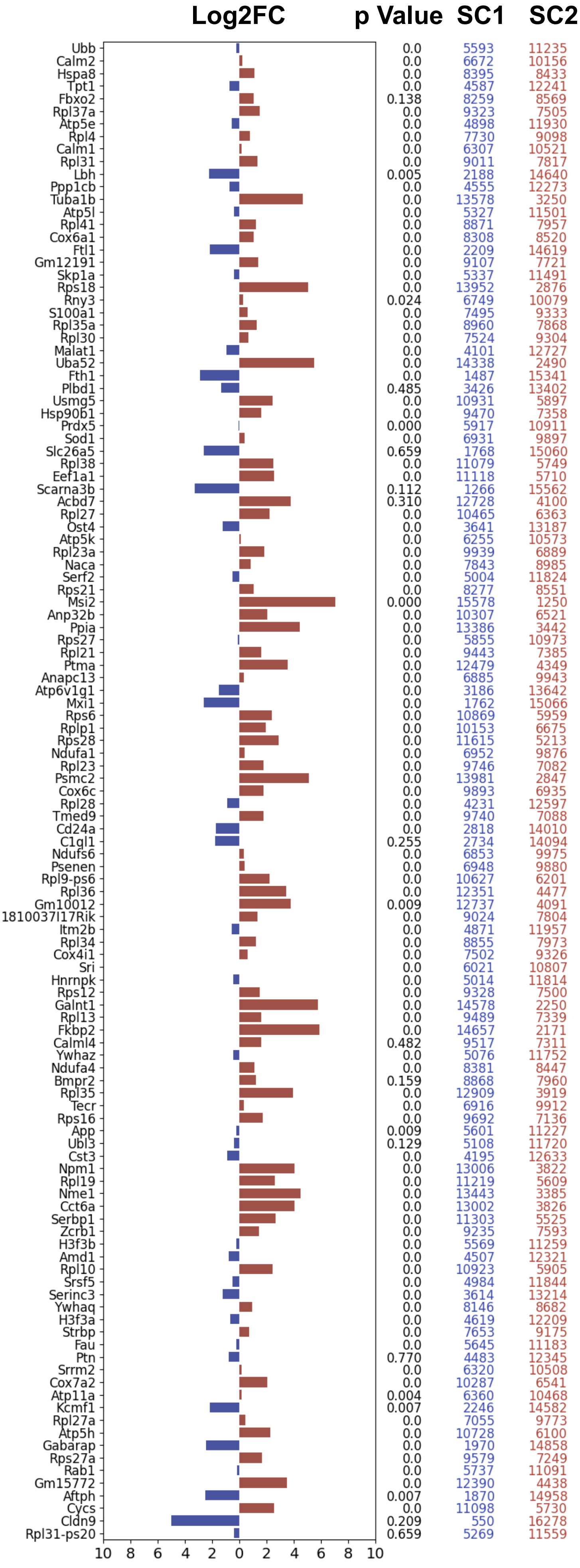

Gene	p Value	SC1	SC2
Ubb	0.0	5593	11235
Calm2	0.0	6672	10156
Hspa8	0.0	8395	8433
Tpt1	0.0	4587	12241
Fbxo2	0.138	8259	8569
Rpl37a	0.0	9323	7505
Atp5e	0.0	4898	11930
Rpl4	0.0	7730	9098
Calm1	0.0	6307	10521
Rpl31	0.0	9011	7817
Lbh	0.005	2188	14640
Ppp1cb	0.0	4555	12273
Tuba1b	0.0	13578	3250
Atp5l	0.0	5327	11501
Rpl41	0.0	8871	7957
Cox6a1	0.0	8308	8520
Ftl1	0.0	2209	14619
Gm12191	0.0	9107	7721
Skp1a	0.0	5337	11491
Rps18	0.0	13952	2876
Rny3	0.024	6749	10079
S100a1	0.0	7495	9333
Rpl35a	0.0	8960	7868
Rpl30	0.0	7524	9304
Malat1	0.0	4101	12727
Uba52	0.0	14338	2490
Fth1	0.0	1487	15341
Plbd1	0.485	3426	13402
Usmg5	0.0	10931	5897
Hsp90b1	0.0	9470	7358
Prdx5	0.000	5917	10911
Sod1	0.0	6931	9897
Slc26a5	0.659	1768	15060
Rpl38	0.0	11079	5749
Eef1a1	0.0	11118	5710
Scarna3b	0.112	1266	15562
Acbd7	0.310	12728	4100
Rpl27	0.0	10465	6363
Ost4	0.0	3641	13187
Atp5k	0.0	6255	10573
Rpl23a	0.0	9939	6889
Naca	0.0	7843	8985
Serf2	0.0	5004	11824
Rps21	0.0	8277	8551
Msi2	0.000	15578	1250
Anp32b	0.0	10307	6521
Ppia	0.0	13386	3442
Rps27	0.0	5855	10973
Rpl21	0.0	9443	7385
Ptma	0.0	12479	4349
Anapc13	0.0	6885	9943
Atp6v1g1	0.0	3186	13642
Mxi1	0.0	1762	15066
Rps6	0.0	10869	5959
Rplp1	0.0	10153	6675
Rps28	0.0	11615	5213
Ndufa1	0.0	6952	9876
Rpl23	0.0	9746	7082
Psmc2	0.0	13981	2847
Cox6c	0.0	9893	6935
Rpl28	0.0	4231	12597
Tmed9	0.0	9740	7088
Cd24a	0.0	2818	14010
C1ql1	0.255	2734	14094
Ndufs6	0.0	6853	9975
Psenen	0.0	6948	9880
Rpl9-ps6	0.0	10627	6201
Rpl36	0.0	12351	4477
Gm10012	0.009	12737	4091
1810037I17Rik	0.0	9024	7804
Itm2b	0.0	4871	11957
Rpl34	0.0	8855	7973
Cox4i1	0.0	7502	9326
Sri	0.0	6021	10807
Hnrnpk	0.0	5014	11814
Rps12	0.0	9328	7500
Galnt1	0.0	14578	2250
Rpl13	0.0	9489	7339
Fkbp2	0.0	14657	2171
Calml4	0.482	9517	7311
Ywhaz	0.0	5076	11752
Ndufa4	0.0	8381	8447
Bmpr2	0.159	8868	7960
Rpl35	0.0	12909	3919
Tecr	0.0	6916	9912
Rps16	0.0	9692	7136
App	0.009	5601	11227
Ubl3	0.129	5108	11720
Cst3	0.0	4195	12633
Npm1	0.0	13006	3822
Rpl19	0.0	11219	5609
Nme1	0.0	13443	3385
Cct6a	0.0	13002	3826
Serbp1	0.0	11303	5525
Zcrb1	0.0	9235	7593
H3f3b	0.0	5569	11259
Amd1	0.0	4507	12321
Rpl10	0.0	10923	5905
Srsf5	0.0	4984	11844
Serinc3	0.0	3614	13214
Ywhaq	0.0	8146	8682
H3f3a	0.0	4619	12209
Strbp	0.0	7653	9175
Fau	0.0	5645	11183
Ptn	0.770	4483	12345
Srrm2	0.0	6320	10508
Cox7a2	0.0	10287	6541
Atp11a	0.004	6360	10468
Kcmf1	0.007	2246	14582
Rpl27a	0.0	7055	9773
Atp5h	0.0	10728	6100
Gabarap	0.0	1970	14858
Rps27a	0.0	9579	7249
Rab1	0.0	5737	11091
Gm15772	0.0	12390	4438
Aftph	0.007	1870	14958
Cycs	0.0	11098	5730
Cldn9	0.209	550	16278
Rpl31-ps20	0.659	5269	11559

Supplemental Figure S22. Comparison to Deiters cells (Ranum et al. 2019)

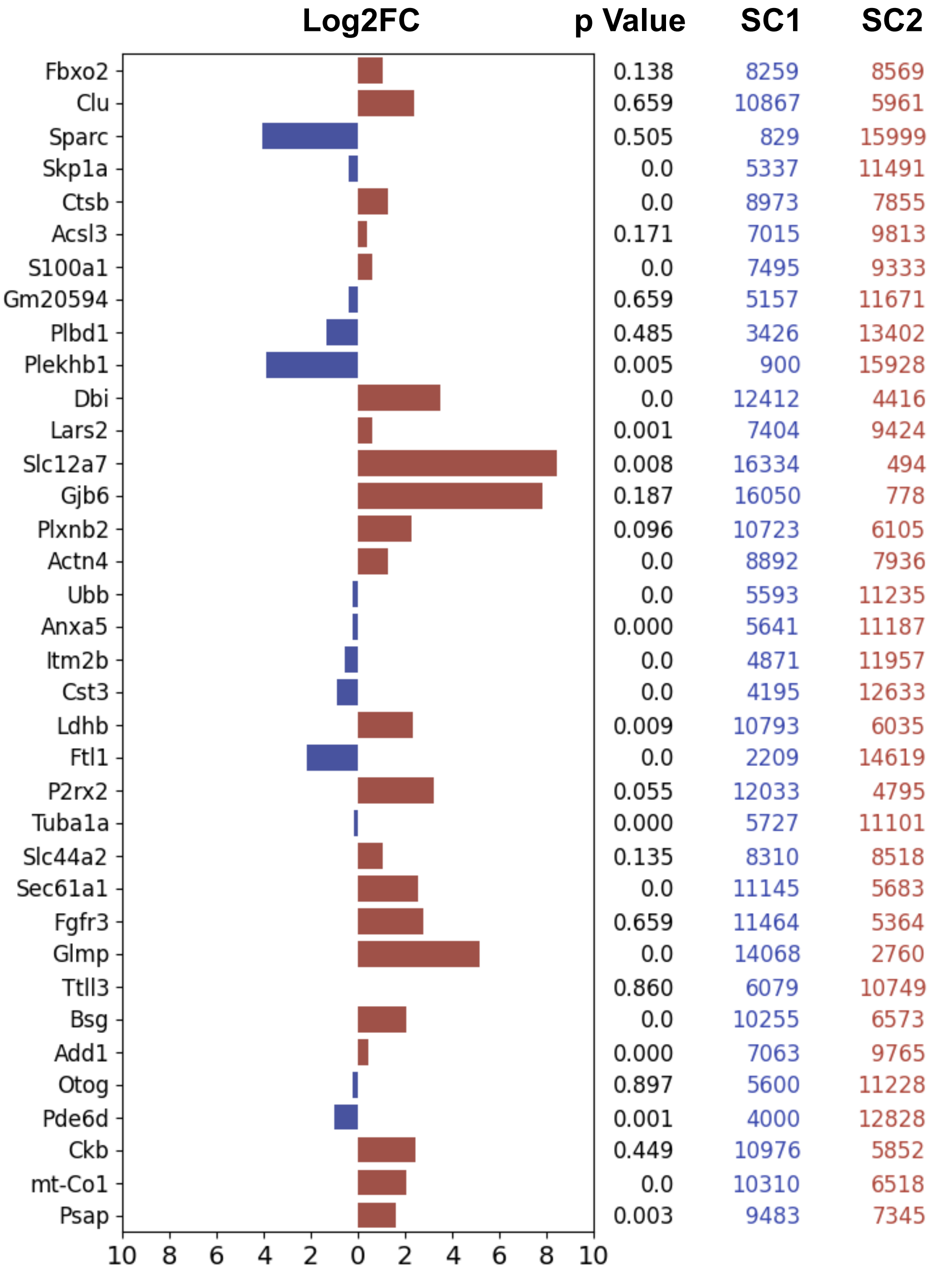

Gene	p Value	SC1	SC2
Fbxo2	0.138	8259	8569
Clu	0.659	10867	5961
Sparc	0.505	829	15999
Skp1a	0.0	5337	11491
Ctsb	0.0	8973	7855
Acsl3	0.171	7015	9813
S100a1	0.0	7495	9333
Gm20594	0.659	5157	11671
Plbd1	0.485	3426	13402
Plekhb1	0.005	900	15928
Dbi	0.0	12412	4416
Lars2	0.001	7404	9424
Slc12a7	0.008	16334	494
Gjb6	0.187	16050	778
Plxnb2	0.096	10723	6105
Actn4	0.0	8892	7936
Ubb	0.0	5593	11235
Anxa5	0.000	5641	11187
Itm2b	0.0	4871	11957
Cst3	0.0	4195	12633
Ldhb	0.009	10793	6035
Ftl1	0.0	2209	14619
P2rx2	0.055	12033	4795
Tuba1a	0.000	5727	11101
Slc44a2	0.135	8310	8518
Sec61a1	0.0	11145	5683
Fgfr3	0.659	11464	5364
Glmp	0.0	14068	2760
Ttll3	0.860	6079	10749
Bsg	0.0	10255	6573
Add1	0.000	7063	9765
Otog	0.897	5600	11228
Pde6d	0.001	4000	12828
Ckb	0.449	10976	5852
mt-Co1	0.0	10310	6518
Psap	0.003	9483	7345

Supplemental Figure S23. Comparison to IHC (Ranum et al. 2019)

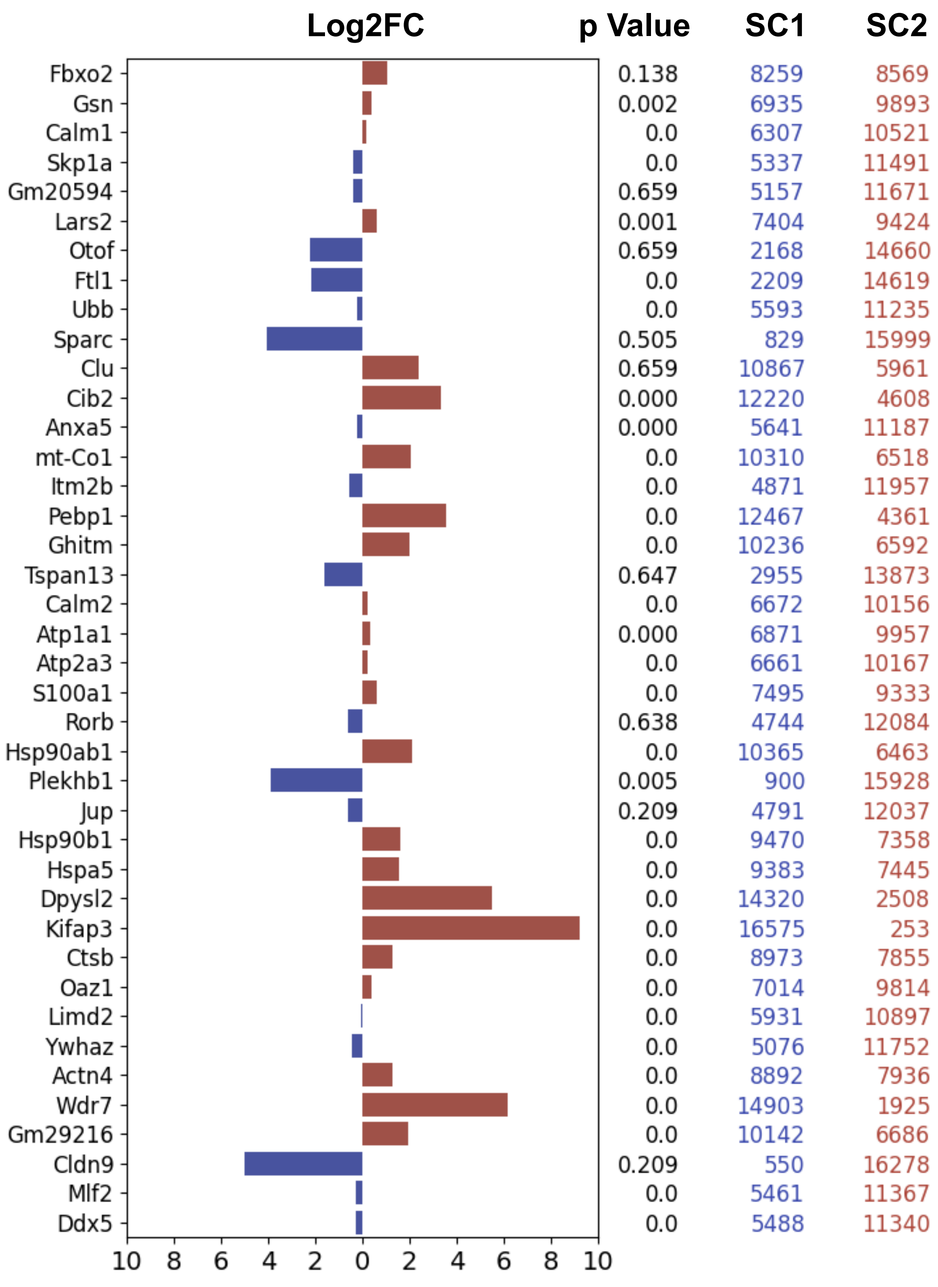

Supplemental Figure S24. Comparison to OHC (Ranum et al. 2019)

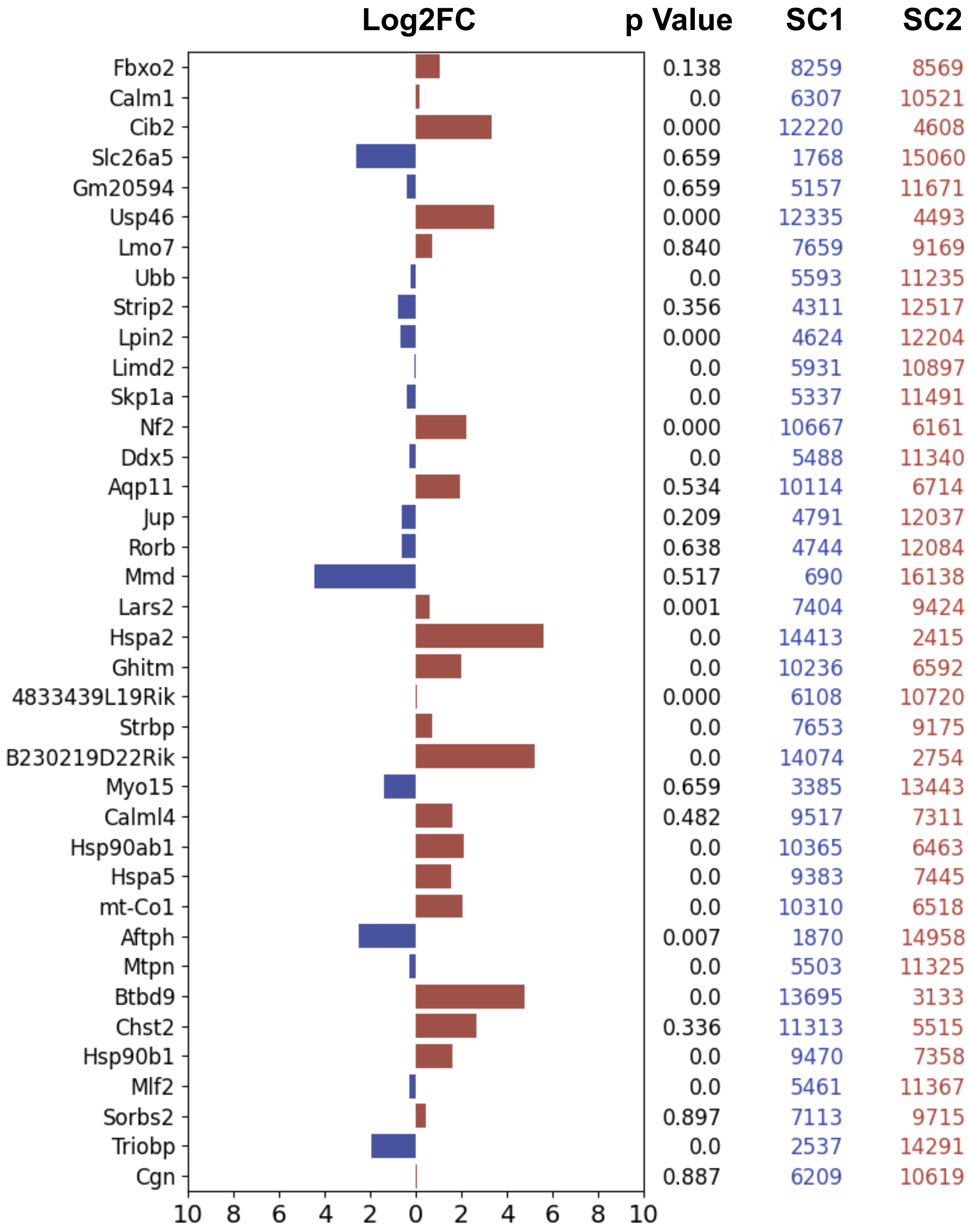

Gene	p Value	SC1	SC2
Fbxo2	0.138	8259	8569
Calm1	0.0	6307	10521
Cib2	0.000	12220	4608
Slc26a5	0.659	1768	15060
Gm20594	0.659	5157	11671
Usp46	0.000	12335	4493
Lmo7	0.840	7659	9169
Ubb	0.0	5593	11235
Strip2	0.356	4311	12517
Lpin2	0.000	4624	12204
Limd2	0.0	5931	10897
Skp1a	0.0	5337	11491
Nf2	0.000	10667	6161
Ddx5	0.0	5488	11340
Aqp11	0.534	10114	6714
Jup	0.209	4791	12037
Rorb	0.638	4744	12084
Mmd	0.517	690	16138
Lars2	0.001	7404	9424
Hspa2	0.0	14413	2415
Ghitm	0.0	10236	6592
4833439L19Rik	0.000	6108	10720
Strbp	0.0	7653	9175
B230219D22Rik	0.0	14074	2754
Myo15	0.659	3385	13443
Calml4	0.482	9517	7311
Hsp90ab1	0.0	10365	6463
Hspa5	0.0	9383	7445
mt-Co1	0.0	10310	6518
Aftph	0.007	1870	14958
Mtpn	0.0	5503	11325
Btbd9	0.0	13695	3133
Chst2	0.336	11313	5515
Hsp90b1	0.0	9470	7358
Mlf2	0.0	5461	11367
Sorbs2	0.897	7113	9715
Triobp	0.0	2537	14291
Cgn	0.887	6209	10619
